# Machine Learning-Guided Video Analysis Identifies Sound-Evoked Pain-Related Behaviors from Facial Grimace and Body Cues in Mice

**DOI:** 10.1523/ENEURO.0064-26.2026

**Published:** 2026-09-01

**Authors:** Benjamin J. Seicol, Amelie Valles-Jane, Anna Kohler, Elisabeth Glowatzki, Megan B. Wood

**Affiliations:** ^1^The Center for Hearing and Balance, The Johns Hopkins School of Medicine, Baltimore, Maryland 21205; ^2^Department of Otolaryngology Head and Neck Surgery, The Johns Hopkins School of Medicine, Baltimore, Maryland 21205; ^3^Department of Neuroscience, The Johns Hopkins School of Medicine, Baltimore, Maryland 21205; ^4^Department of Hearing & Speech Sciences, Vanderbilt School of Medicine, Nashville, Tennessee 37232

**Keywords:** cochlea, deep learning, facial grimace, hyperacusis, migraine

## Abstract

Humans can experience sound-evoked pain, either from extremely loud sounds or in cases of pain hyperacusis from typically tolerable sounds. However, the mechanisms underlying sound-evoked pain remain poorly understood. Developing behavioral methods to measure sound-evoked pain in animal models is critical for elucidating these mechanisms. Here, a machine learning-based approach was developed to measure sound-evoked pain in freely moving mice of both sexes by analyzing facial grimace and body position from video recordings during sound exposure. Facial grimace, a commonly used method to detect pain in mice, and body position, which can be used to measure postural and movement changes that also indicate pain, were both quantified using a deep neural network model trained to extract established facial and body features from video recorded by a single camera. To validate the model's capability to detect pain, a known painful state, migraine induced by the injection of the neuropeptide calcitonin gene-related peptide (CGRP), was used. Using this machine learning-based approach, the ability to quantify a pain response from CGRP-induced migraine, distinct from baseline behavior, was demonstrated, resulting in a defined pain threshold. Sound exposures at high intensities elicited significant changes in facial grimace and body position, in comparison, surpassing the pain threshold calculated from CGRP-induced migraine. These behavioral changes were absent in *Tmie*-knock-out mice, which lack functional sound transduction in the cochlea. This automated, high-throughput framework enables objective and sensitive analysis of pain-related behavior providing a foundation for future studies investigating the peripheral and central mechanisms of sound-evoked pain.

## Significance Statement

This study introduces a quantitative framework for assessing pain using a single-camera setup and machine learning-guided analysis to capture and analyze mouse behavior. By integrating two established pain metrics, facial grimace and attenuated movement, this method enables precise, noninvasive quantification of pain-related behaviors. The approach was validated with a well-characterized pain model, migraine, induced by injection of the neuropeptide calcitonin gene-related peptide, demonstrating the ability to quantify a pain response distinct from baseline behavior. By applying this framework to sound-evoked pain, the data revealed that exposure to intense sound triggers significant pain behavioral responses. These novel findings provide insights into the behavioral manifestations and neural underpinnings of sound-evoked pain, offering a robust tool for studying the mechanisms of pain perception.

## Introduction

Humans can experience pain in response to sound. Such pain can either occur as a reaction to loud sounds (>120 dB SPL; von Gierke and Brown, 1953; Frank and Kössl, 1996; Sherlock and Formby, 2005; Manohar et al., 2020) or, in the case of pain hyperacusis, can be a response to typically tolerable noise exposures (Pienkowski et al., 2014; Tyler et al., 2014; Pollard, 2019). When sound exposure becomes painful, the mechanisms that lead to sound-evoked pain remain unknown (Salvi et al., 2022; Jahn et al., 2025). Processing of sound-evoked pain information, for example, may involve activation of brain regions canonically associated with pain, such as the thalamus, along with paradoxical hypoactivity in the auditory cortex (Song et al., 2014; Zhou et al., 2022; Clayton et al., 2024). Developing sensitive behavioral methods to measure sound-evoked pain in laboratory animals will help to determine whether animal models recapitulate human pathology and allow further investigation of peripheral and central mechanisms responsible for sound-evoked pain(Radziwon and Salvi, 2020; Salvi et al., 2022; Smith et al., 2025).

Behavioral reflexes in response to sound onset are widely used to assess animals' auditory sensitivity, for example, by the acoustic startle response (Hickox and Liberman, 2014; Salloum et al., 2016; Manohar et al., 2020). Broadly, pain can be measured in mice either by (1) withdrawal reflex, such as to thermal or mechanical stimuli (Deuis et al., 2017), (2) operant conditions of forced-choice tasks (Sufka, 1994), or (3) assessment of behavioral signatures associated with affective pain [e.g., facial grimace (Zhang et al., 2022), movement (Bohic et al., 2023)]. During pain perception, mice “grimace,” like humans (Langford et al., 2010; McCoy et al., 2024), and therefore pain can be quantified by characteristic changes in their facial features, for example, by eyes squinting (Le Moëne and Larsson, 2023). Facial grimace has effectively been used to measure painful states in animal models, for example, in the case of migraine (Rea et al., 2018). Automated facial grimace detection and body movement analysis of persistent pain in mice, including postoperative and migraine pain, has proven comparable to individual investigators scoring behavioral cues; it has reduced bias and allowed for high-throughput analysis of large datasets (Tuttle et al., 2018; Chiang et al., 2022; Bohic et al., 2023). Body movement has also been analyzed parallel, however, measuring behavioral changes in facial grimace and body position has required two or more camera angles (en face or profile and from above; Cobos et al., 2012; Bohic et al., 2023).

Due to the distinct behavioral approaches used to measure pain in animals, different types of pain are often defined as either “reflexive” or “spontaneous,” where reflexive pain usually refers to a withdrawal reflex to an acute external sensory stimulus. In contrast, spontaneous pain is typically used to describe ongoing painful states (Bennett, 2012). Pain evoked by sound exposures that persists for periods of time on the order of minutes is, therefore, a type of spontaneous pain (herein now referred to as “persistent pain”). Here, a deep neural network was trained to extract formerly established facial features (Le Moëne and Larsson, 2023) from video recordings of freely moving mice exposed to various levels of sound intensity presentations. Moreover, body position measurements from the same camera angle as facial grimace recordings were added to the analysis to simultaneously detect changes in mouse posture and locomotion behaviors associated with pain (Turner et al., 2019). This approach of analyzing combined facial grimace and body posture was validated with a well-characterized pain model, migraine, induced by injection of the neuropeptide CGRP (calcitonin gene-related peptide), which demonstrated the ability to quantify a pain response distinct from baseline behavior and define a pain threshold. Sound exposure at high intensities elicited significant changes in facial grimace and body posture and, in comparison, surpassed the pain threshold established during migraine validation. Such changes did not occur in *Tmie*-knock-out (*Tmie^−/−^)* mice that lack normal cochlear transduction of sound (Gleason et al., 2009; Wu et al., 2017; Holt et al., 2021; Fettiplace et al., 2022) validating that normal cochlear transduction is needed to induce sound-evoked pain-related behavior in mice. Facial grimace measurements combined with body position information extracted by deep learning from video recordings of mice presented with sound allowed for sensitive, objective, and high-throughput analysis of mouse sound-evoked pain.

## Materials and Methods

### Animals

Six-week-old C57BL6/J mice of both sexes were used. Both sexes were tested separately. At the time of testing, animals were group-housed in a low noise vivarium. The housing room was maintained at 12:12 h light/dark cycle (7:00–19:00). Up to five mice were housed per one filter top shoebox cage (30 × 19 × 13 cm^3^). All procedures were approved by the Animal Care and Use Committee and follow the National Institutes of Health ARRIVE Guidelines.

### Behavioral testing setup design

Individual polycarbonate containers (testing chamber; interior dimensions: 9 × 5 × 5 cm) were fabricated onsite and placed in an acoustic chamber prior to testing. The acoustic chamber was lined with LED lights for filming. At all times, the mice were awake and freely moving. Mice were oriented to maintain a profile position via scent cues from air holes positioned at one end of their polycarbonate containers (Ernst et al., 2020). When multiple mice were recorded simultaneously, two mice of the same sex were placed individually into two chambers oriented to face opposite each other while maintaining a profile position via scent cues from air holes positioned at opposite ends of each container. The mice were prevented from seeing each other by adding a white opaque barrier between the containers. A GoPro Hero 11 Black was centered to capture mice during the protocol. Overhead speakers were fitted into the sound chamber to allow for sound exposure.

### Video editing

Sound exposure videos were ∼26 min in length and were filmed in 4K resolution at 30 frames/s. Videos were cropped to ∼1,000 × 1,500 pixels (height vs width) to analyze one of two mice at a time when multiple mice were recorded simultaneously. The recordings from the CGRP- and saline-injected mice yielded one 10 min and one 20 min video filmed in 4K resolution at 30 frames/s. Each video was then cropped using iMovie to focus on a single mouse at a time for the most accurate labeling for each condition. Time segments (epochs) of video were cropped to the desired length to cover a given condition (e.g., CGRP peak epoch, 110 dB SPL, baseline, etc.)

### Pose estimation using DeepLabCut

**Figure 1.**
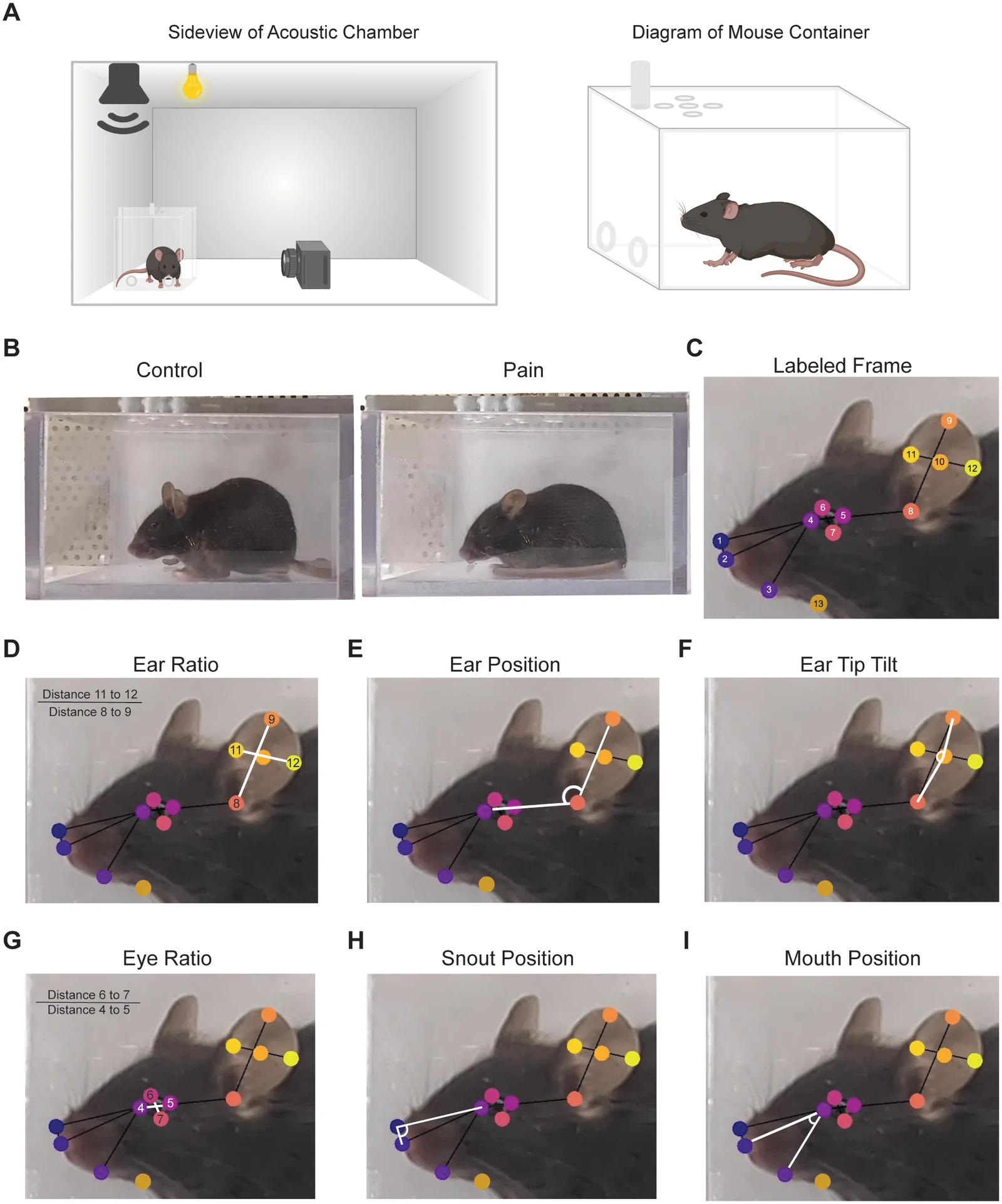
Detection of facial grimace parameters in freely moving mice using a deep neural network ***A***, Left, A schematic of the inside of the acoustic booth used for facial grimace video recording. Right, A diagram of the custom-built enclosure from the view of the camera. The ovals on the left wall and top indicate scent holes. Figure not to scale. ***B***, Labeled frames from the same video of a mouse in a control condition (left) and a painful condition (right). ***C***, A labeled frame of a mouse face showing the 13 markers on the face (numbered, colored dots). The black lines are skeletons generated by the model. Colored dot 1, Nose Tip (upper tip of nose); 2, Nose Bot (bottom of nose); 3, mouth; 4, inner eye (anterior edge of eye); 5, Outer Eye (caudal edge of eye); 6, Eye Up (highest point of eye lid on dorsal edge); 7, Eye Down (lowest point of eyelid on ventral edge); 8, Ear Canal (edge of hair and ear canal); 9, Ear Tip (edge of pinna furthest from the ear canal); 10, Ear Center (center of ear pinna between Points 9, 10, 11, and 12); 11, Ear Med (halfway up the pinna on the medial side); 12, Ear Lat (halfway up the pinna on the lateral side of the ear); 13, Neck (point where the head connects to the body). ***D***, Ear Ratio was calculated as the ratio of the length of the lines in pixels from Ear Med to Ear Lat (Point 11 to 12) and Ear Canal to Ear Tip (Point 8 to 9) indicated by white lines. ***E***, Ear Position was calculated as the obtuse angle formed by lines connecting Inner Eye, Ear Canal, and Ear Tip with Ear Canal as the vertex as indicated in white. ***F***, Ear Tip Tilt was calculated as the angle formed by lines connecting Ear Canal to Ear Center to Ear Tip with Ear Center as the vertex as indicated in white. ***G***, Eye Ratio was calculated as the ratio of the length of the lines in pixels from Eye Up to Eye Down (Point 6 to 7) and Inner Eye to Outer Eye indicated by white lines (Point 4 to 5). ***H***, Snout Position was calculated as the acute angle formed by lines connecting Nose Tip, Inner Eye, and Nose Bottom with Inner Eye as the vertex as indicated in white. ***I***, Mouth Position was calculated as the acute angle formed by lines connecting Nose Bottom, Inner Eye, and Mouth with Inner Eye as the vertex as indicated in white.

The deep neural network was trained to place thirteen markers on the face in each video frame where the face is in profile using DeepLabCut (DLC; Mathis et al., 2018; Nath et al., 2019). The 13 markers were labeled inner eye, outer eye, eye up, eye down, nose tip, nose bottom, mouth, ear canal, ear tip, ear center, ear medial, ear lateral, and neck (Fig. 1; Le Moëne and Larsson, 2023). DLC was installed on a Windows OS computer with an Intel Core i5-7600K CPU at 3.80 GHz processor and an Nvidia GeForce GTX 1070 graphics card. ResNet-50–based neural network with default parameters was run for up to 800,000 iterations of training on 816 annotated frames [far above the 200 frames recommended by Mathis et al. (2018) and Nath et al. (2019)] from all cohorts of animals. The dataset was split as 95% for training and 5% for testing. After training, annotated images were visually inspected to ensure the markers were placed on the appropriate key points of the body and face. Data for new videos analyzed were filtered based off a >0.95 likelihood (*p*-cutoff ≥0.95). A lower limit of at least 30 analyzed frames per video analyzed was used to consider that epoch valid for statistical comparisons, and all recorded time periods fit this criterion. The average RMSE of all pose markers using a *p*-cutoff at 0.95 was 3.6 pixels. A median number 18,968 frames was analyzed per video.

### Defining face and position parameters

A representative annotated frame showing DLC-generated pose estimated points is shown in Figure 1. Lines between points (skeletons) were created automatically in DLC (Fig. 1*A*). The skeletons used were nose tip to nose bottom, mouth to inner eye, nose bottom to inner eye, inner eye to outer eye, eye up to eye down, inner eye to ear canal, ear canal to ear tip, and medial ear to lateral ear. These skeletons are used to define eye ratio, ear ratio, ear angle, ear position, snout position, and mouth position. Representative images show changes in these parameters between control and pain conditions (Fig. 1*B*). Angles between certain skeletons and ratios of certain skeletons were used to generate measurements of facial parameters as follows: Ear Ratio was calculated as the ratio of the length of the lines in pixels from Ear Med to Ear Lat and Ear Canal to Ear Tip indicated by white lines (Fig. 1*C*). Ear Position was calculated as the obtuse angle formed by lines connecting Inner Eye, Ear Canal, and Ear Tip with Ear Canal as the vertex as indicated in white (Fig. 1*D*). Ear Tip Tilt was calculated as the angle formed by lines connecting Ear Canal to Ear Center to Ear Tip with Ear Center as the vertex as indicated in white (Fig. 1*E*). Eye Ratio was calculated as the ratio of the length of the lines in pixels from Eye Up to Eye Down and Inner Eye to Outer Eye indicated by white lines (Fig. 1*F*). Snout Position was calculated as the acute angle formed by lines connecting Nose Bottom, Nose Tip, and Inner Eye with Nose Tip as the vertex as indicated in white (Fig. 1*G*). Mouth Position was calculated as the acute angle formed by lines connecting Nose Bottom, Inner Eye, and Mouth with Inner Eye as the vertex as indicated in white (Fig. 1*H*). The training and likelihood cut off prevented inclusion of measurements of the ear and eye affected by the animal being in a suboptimal presentation during movement. However, the Mouth Position and Snout Position measurements were affected by subtle changes to animal movement. To counteract artifacts based on suboptimal mouse position, the limits to the angles reported by Le Moëne and Larsson were used as ground truth (Le Moëne and Larsson, 2023). Thus, Mouth Position was reported when the likelihood for the Mouth marker was above 0.95, and the angle was between 10 and 50°. Snout Position was recorded when the likelihood was above 0.95 for the Nose Tip and the angle was between 50 and 110°.

The Relative Nose Tip Position was the distance in pixels that the nose tip point was below the (0,0) coordinate in the top left most corner of the chamber, with increasingly negative values representing the mouse's nose tip at a low place in the chamber (Fig. 3*A*,*B*). The nose tip pose estimated point was further used to calculate the percentage of analyzed frames where this point is above a horizontal line drawn at −450 pixels (Fig. 3*C*; corresponding to the back of the mouse when all four paws are planted on the chamber floor, “Percentage of Nose Tip in Top 1/3 of Chamber”) and to count the incidences when the nose tip crosses a vertical line drawn at 900 pixels (Fig. 3*D*; deliberately set off-center to ensure full turns of the mouse were required to count as an incidence of crossing the vertical line, “Vertical Line Crosses of Nose Tip”) . These measurements were calculated as the percentage of frames above horizontal line and total number of vertical line crosses, respectively. The horizontal line in Percentage of Nose Tip in Top 1/3 of Chamber was set at −450 because the *y*-axis pixel count varied from ∼900–1,000 based on cropping of the video. The walls of the chamber (top and bottom, including space outside the chamber walls) accounted for 150–300 pixels of that space leaving the interior of the chamber at an average of 900 pixels. Therefore, one-third of the chamber is 300, but 150 was added to account for the top chamber wall, thus −450. The Percentage of Nose Tip in Top 1/3 of Chamber provides a gross approximation of decreased rearing, exploration of the top portion of the chamber, and climbing behaviors. While Vertical Line Crosses of Nose Tip capture how often the mouse turns inside the chamber, which is influenced by exploration behavior and total locomotion. Finally, Face Inclination was calculated as the percentage of analyzed frames where the nose tip was below the neck (Fig. 3*E*). Mice in pain tend to hunch forward causing the nose tip to be below the neck point; therefore this measure of face inclination would increase the more the mouse was hunched forward. A minimum of 30 valid frames per parameter from a condition within a video was required for the data to be included.

### CGRP or saline injections

Mice were placed individually into the testing chamber and allowed 10 min of acclimation to the container. Ten minutes of video was subsequently recorded to measure baseline behavior for each mouse. Mice were then removed from the container and injected with either CGRP (100 µg/ml in saline, i.p.) or saline (vehicle) and immediately returned to the container. Since this dose of CGRP induces a migraine that peaks 30 min after injection (Rea et al., 2018), the video was recorded from 20 to 40 min postinjection. In total, 30 min of video was recorded for each mouse and was subsequently segmented for analysis as described below.

### Sound exposure protocol

Mice received an acclimation period of 30 min to their containers the day before the sound exposure protocol. Calibrations within the acoustic chamber were completed each day before noise exposure took place to ensure exposures were accurate and consistent. The two readings checked were 90 and 120 dB SPL. The calibration consisted of taking readings from the corner of each container and the center of the acoustic chamber using a sound level meter model LxT2 (Larson Davis). Mice were placed into the same individual polycarbonate containers (described above) within an acoustic chamber fitted with overhead speakers. The mice were unable to look at each other throughout the exposure to eliminate visual cues from the other mouse. The video was centered in the middle capturing both mice during sound exposures. The sound exposure protocol began with 10 min without sound played from the speakers; then sound was presented in an interleaved protocol ranging from 70 to 120 dB SPL of broadband (2–20 kHz, a broad bandwidth was used to lower cochlear damage potential). To prevent acoustic startle responses and audiogenic seizure, sound was ramped on and off at the beginning and end of the 2 min periods over the course of 3 s (Cory and Ison, 1979). The sound was played for 2 min followed by 2 min of no sound after each sound presentation. The 2 min period without sound (from 8 to 10 min in each video) was used as the control for each mouse. Sound presentation was interleaved in the following order: 90, 110, 70, 100, 80, and 120 dB SPL.

### Data analysis and statistics

Filtered CSV files were output once DLC finished analyzing each video. These files were converted into xlsx files to be quantified. Pairwise comparisons, such as *Baseline* versus All – CGRP, were tested using paired, two-tailed, Student’s *t* tests. Statistical differences for groups with repeated-measures were assessed using repeated-measures ANOVA for balanced groups and repeated-measures mixed-effects model for subgroups tested in longitudinal designs (e.g., CGRP baseline and subgroups within migraine and sound exposure epochs) and post hoc Tukey's correction for multiple comparisons unless noted. Effect sizes are shown as Cohen's *d*. For any Wilcoxon rank-sum comparisons when the data were not normal, the effect size is reported as a rank biserial correlation (*r*). Mice of both sexes were used, but sex differences were not examined due to the small cohort of animals.

## Results

Detecting persistent pain in mice has been performed before by analyzing facial grimace (Langford et al., 2010; Tuttle et al., 2018; Chiang et al., 2022; Le Moëne and Larsson, 2023; McCoy et al., 2024). Here, facial grimace detection has been adapted from work that has used six defined features of the face from the profile of a mouse's face from videos of freely moving mice (Le Moëne and Larsson, 2023). It was further possible to expand beyond facial grimace using the same videos, and some of these same markers to analyze body position information of the mice. Average values of each facial grimace parameter and separately of four specific body position parameters were calculated within defined periods (epochs) and combined to create an inventory of pain-related behavior. This inventory was validated, and pain-related behavior thresholds were determined, based on a known painful stimulus (CGRP-induced migraine). This approach was subsequently used to test the level of sound that elicits changes in behavior associated with pain in mice. Additionally, mice without normal cochlear function were used to determine whether sound transduction in the inner ear is necessary for the generation of sound-evoked pain- related behavior.

### Detection of facial grimace parameters in freely moving mice using a deep neural network

A deep neural network was trained to measure changes in facial grimace by automatic placement of 13 points on the face of mice with skeletons connecting these points from video recordings of mice performed in controlled conditions (Fig. 1). This approach allowed for high-throughput analysis of thousands of video frames from hours of recordings, greatly reducing the analysis time compared with manual annotation by blind observers as done before with the mouse grimace scale (Langford et al., 2010). In this study, facial grimace measurements were taken from mice enclosed in a custom-built clear chamber with scent holes on only one side meant to encourage mice to remain mostly in profile with respect to the camera (Fig. 1*A*). Representative images of mice displaying pain-related behavior versus control conditions illustrate the changes in the face, for example, facial grimace of a mouse in a pain state manifests with clear changes in ear positions and eye squinting, as well as subtle changes in snout and mouth positions (Fig. 1*B*). Changes can also be seen in body position, such as the mouse being hunched over and the head angle pointing down, which will be described in detail below. Based on the video recordings, six facial parameters (Le Moëne and Larsson, 2023) were calculated from the pose estimated points on the mouse's face (Fig. 1*C*).

**Figure 2.**
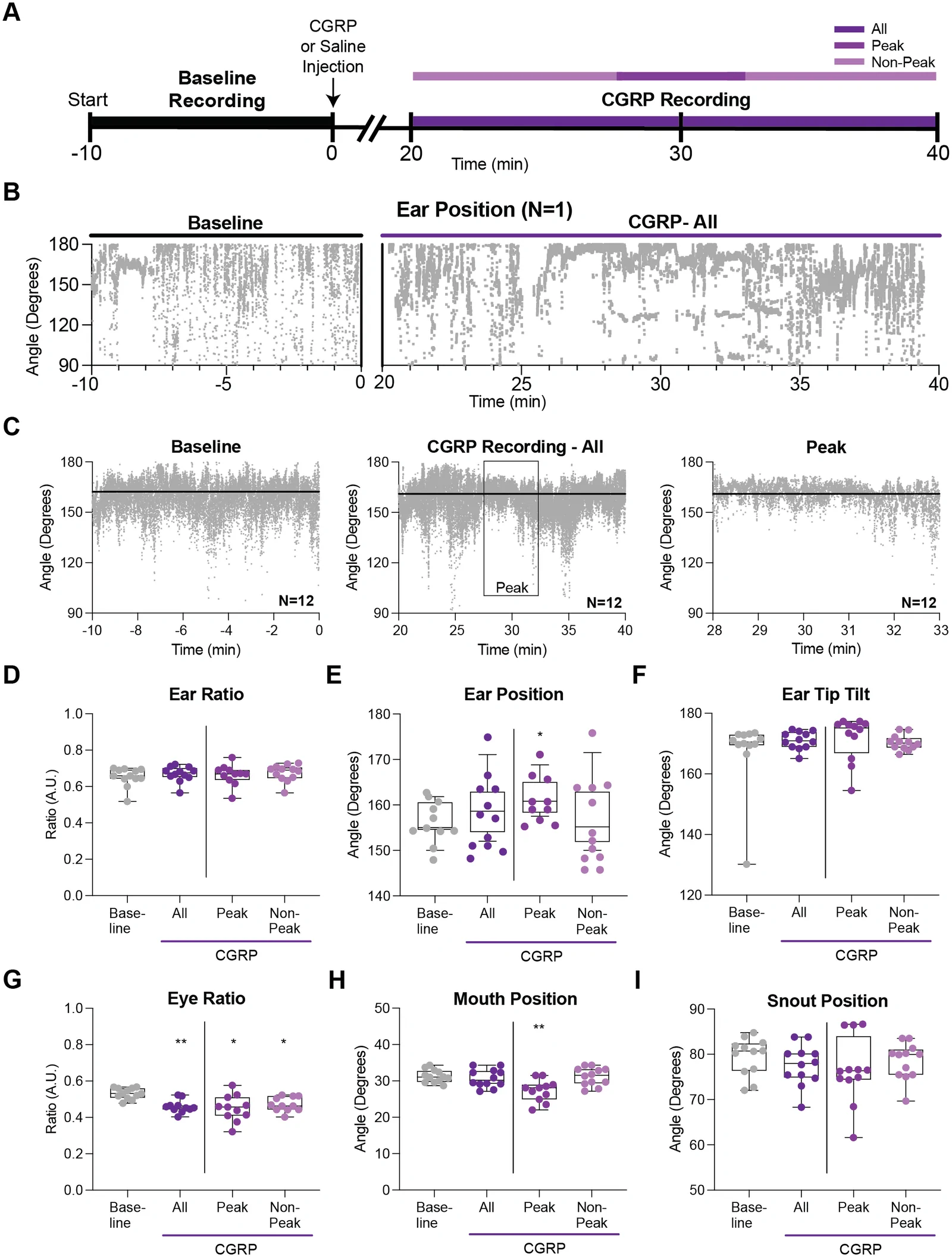
Facial grimace scores in mice that experience migraine after CGRP injection. ***A***, Timeline of CGRP injection experiment. Animals were filmed for a 10-minute baseline period. CGRP was injected based on body weight. After a 10 min period, mice were filmed for 20 min surrounding the peak of action CGRP which occurs 30 min after injection. ***B***, Example of the Ear Position data from the baseline and CGRP time periods of one mouse (*N* = 1). Each dot is the measurement from a single frame of video. ***C***, Averaged data for each frame of video for Ear Position from 12 mice (*N* = 12) during *Baseline* and the entire CGRP Recording. The right graph shows the average Ear Position for 12 mice during the peak time period as indicated in ***A*** and the box in the middle graph. The black horizontal line is the median of the baseline time period as a reference in all graphs. ***D–I***, The following graphs show the averaged data for each facial parameter indicated. Each dot is the averaged data for the indicated time period for one mouse. *Baseline* is the averaged data for each mouse for all frames from the *Baseline* video (10 min). “*All*” is the averaged data for each mouse for all frames after CGRP injection. “*Peak*” is the averaged data from minutes 27–31 and after CGRP injection. “*Non-Peak*” is the averaged data from time outside of the peak time period (minutes 20–26 and minutes 32 to 40). Box and whiskers have a line at the median and the box is the interquartile range with the whiskers extending to the maximum and minimum value of the data. ***D***, Ear Ratio. ***E***, Ear Position. ***F***, Ear Tip Tilt. ***G***, Eye Ratio, *Baseline* compared with *All*, paired, two-tailed *t* test; **p = 0.0012; repeated-measures mixed-effects model; **p = 0.0025; post hoc comparisons using Tukey's correction; *Baseline* compared with *Peak*; **p = 0.0215; *Baseline* compared with *Non-Peak*; *p = 0.0111. ***H***, Mouth Position, repeated-measures mixed-effects model; ****p = <0.0001; post hoc comparisons using Tukey's correction; *Baseline* compared with *Peak*; **p = 0.0015; *Peak* compared with *Non-Peak*; *p* = 0.0026. ***I***, Snout Position. If no statistics are noted, data were not significantly different. For extended data showing facial grimace scores from saline-treated mice see Extended Data Figure 2-1.

**Figure 3.**
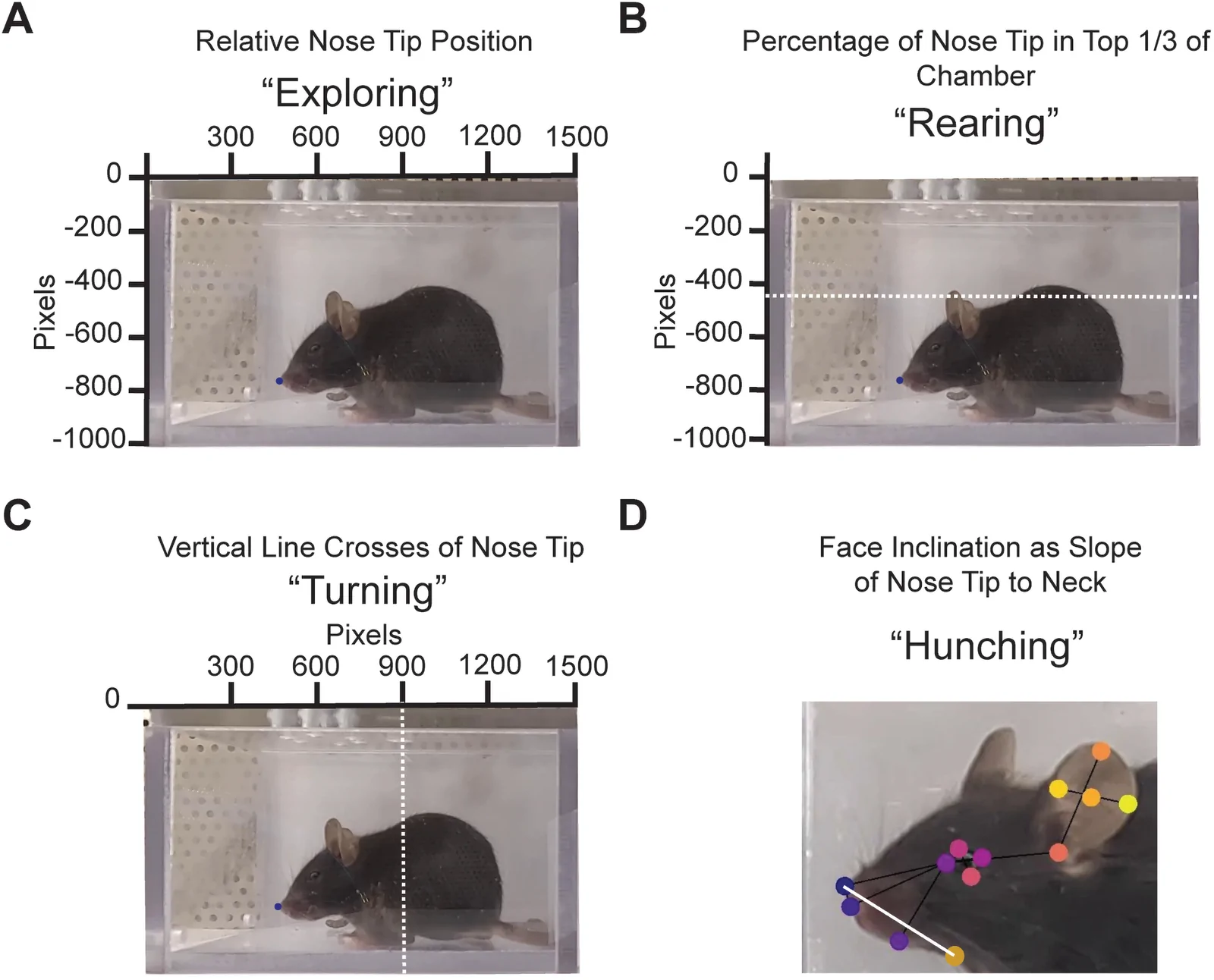
Detection of body position parameters in freely moving mice using a deep neural network. ***A***, Example of a full frame of video of a control mouse with *x*- and *y*-axes showing pixel values. The nose tip is indicated with a navy-blue dot. The *y*-axis pixel value of the nose tip was recorded for each frame and averaged over discrete time periods to create the measurement named Relative Nose Tip Position as a proxy for exploring behavior. ***B***, Example of a full frame of video of a control mouse. The white dashed line at −450 pixels on the *y*-axis indicates the boundary of the top one-third of the chamber. The average number of frames in a time period where the nose tip (navy-blue dot) was above this line based in the *y*-axis was calculated as the Nose Tip Top % measurement as a proxy for rearing behavior. ***C***, Example of a full frame of video of a control mouse. The white dashed line at 900 pixels on the *x*-axis indicates a boundary which was used to count the number of times the nose tip (navy-blue dot) crossed this line in a time period. This was used to calculate the Vertical Line Crosses measurement as a proxy for turning behavior. The line is intentionally offset to capture the full turning of the mouse from one side of the cage to the other instead of rearing straight backward as during grooming. ***D***, Example of a mouse face labeled from the DNN training. The *y*-axis value of the Nose Tip was subtracted from the *y*-axis value of the Neck point (Neck–Nose Tip; indicated by the white line). The Face Inclination measurement was then calculated as the percentage of frames during a time period that the Neck–Nose Tip value was negative meaning the Nose Tip was below the Neck value on the *y*-axis as a proxy for hunched body position.

The DLC software (Mathis et al., 2018; Nath et al., 2019) was used to train a neural network model capable of automatically identifying 13 markers on the mice with skeletons connecting these points to calculate six facial parameters (Fig. 1*D*–*I*). The changes in ear positions were mapped using three parameters: (1) “Ear Ratio” (which goes up as the mouse's ear lies flat against the head thereby increasing the width in relation to the camera in profile view; Fig. 1*D*), (2) “Ear Position” (which measures the angle that increases when the mouse's ear rotates further toward its posterior; Fig. 1*E*), and (3) “Ear Tip Tilt” (which measures the angle of the top half of the ear relative to the bottom half which increases when the ear is flattened against the back of the head; Fig. 1*F*). These three ways of measuring changes in ear position are valuable due to the manner of grimacing in animals that are exaggerated in the ears versus humans (Stubsjøen et al., 2009; Stracke et al., 2011; Schroeer et al., 2024). The three ear-related measures also each reflect a distinct neuromuscular circuit, arising from the (1) transverse auricular muscle, (2) interscutular muscle, and (3) anterior and posterior auricular levators, respectively (Friauf and Herbert, 1985; Heuser and Tenkova, 2020). Additional face grimace parameters measured were “Eye Ratio” (which gets smaller with “eye squinting”; Fig. 1*G*), “Snout Position” (which gets smaller when the angle between the eyes, nose, and mouth is more acute meaning snout is more pointed during pain; Fig. 1*H*), and Mouth Position' (which gets smaller when the bottom lip extends outward similar to clenching of the jaw; Fig. 1*I*).

### Facial grimace scores in mice that experience migraine after CGRP injection indicate pain

Migraine can be elicited by injection of CGRP (Lassen et al., 2002) and is a well-known painful stimulus in mice (Mason et al., 2017). Pain in this model has previously been detected using the mouse grimace scale and peaks at ∼30 min following CGRP administration (Rea et al., 2018). We therefore adopted this model to test our approach of detecting persistent pain with the set of facial grimace parameters described in Figure 1. Mice were injected with CGRP solution (100 µg/kg in saline, i.p.) or injected with saline (vehicle). Video recording began when mice were placed into the chamber for 10 min to record baseline behavior (“*Baseline*”; *t* = −10–0 min; Fig. 2*A*). Mice then received an injection of either CGRP or saline (*t* = 0 min), and the video recording was paused. Video acquisition was restarted 20 min after injection and continued for another 20 min (“*All*”; *t* = 20–40 min) to ensure complete capture of the expected migraine peak (Movie 1). Each facial grimace parameter measured during *Baseline* and *All* can be visualized as a diary plot of all analyzed frames at 30 frames/s, here shown for data from an individual mouse (e.g., Ear Position; Fig. 2*B*). It is notable that there are gaps in the dairy plot illustrating periods where the points could not all be applied to the face, for example, when the mouse was turned away from the camera (Movie 1). Data from all mice (*N* = 12) were superimposed and are shown in Figure 2*C*. The expected peak of migraine-like pain-related behavior from CGRP was anticipated to be around *t* = 30 min. An empirical peak emerged from combined data of facial grimace and body position parameters (quantified in Fig. 5), showing that during a 5 min epoch (“*Peak*”; *t* = 27–32 min) surrounding the expected peak, mice exhibited significant changes in behavior. This empirical peak was therefore analyzed separately from the remaining 15 min of CGRP recording (“*Non-Peak*”), including the time before and after the “*Peak*.”

10.1523/ENEURO.0064-26.2026.f2-1 Figure 2-1 Animals injected with saline do not show changes to facial grimace measurements **A**) These graphs show the averaged data for each facial parameter indicated. Each dot is the averaged data for the indicated time-period for 1 mouse. Baseline is the averaged data for each mouse for all frames from the Baseline video (10 minutes). ‘*All*’ is the averaged data for each mouse for all frames after CGRP injection. ‘Peak’ is the averaged data from minutes 28-33 and after CGRP injection. ‘*Non-Peak*’ is the averaged data from time outside of the peak time period (minutes 20-26 and minutes 32 to 40). Box and whiskers have a line at the median and the box is the interquartile range with the whiskers extending to the maximum and minimum value of the data. Eye Ratio, repeated measures mixed model, p = 0.0465; post hoc comparisons using Tukey’s correction, *Peak* compared to *Non-Peak*, ***, p = 0.0003. **B**) These graphs show the averaged data for each body position measurement indicated. Each dot is the averaged data for 1 mouse. *Baseline* is the averaged data for each mouse for all frames from the Baseline video. ‘*All*’ is the averaged data for each mouse for all frames after CGRP injection. ‘*Peak*’ is the averaged data from minutes 27-31 and after CGRP injection. ‘*Non-Peak*’ is the averaged data from time outside of the peak time period (minutes 20-26 and minutes 32 to 40). Box and whiskers have a line at the median and the box is the interquartile range with the whiskers extending to the maximum and minimum value of the data. If no statistics noted, data was not significantly different. Download Figure 2-1, TIF file.

**Movie 1.** CGRP experiment example. Ten-second video clips from the *Baseline* recording and three segments during the CGRP recording—first 2 min, *Peak*, and last 2 min. The labels were generated by DLC, and any marker above 0.6 likelihood is shown for visualization. Only markers with a likelihood above 0.95 were used for analysis. [View online]

Median values from *Baseline* and *All* epochs were compared using paired, two-tailed, Student’s *t* test for each of the six facial grimace measurements, while *Baseline*, *Peak*, and *Non-Peak* epochs were compared using either repeated-measures one-way ANOVA for balanced groups or repeated-measures mixed-effects model for unbalanced groups (Fig. 2*D*–*I*). Significant differences, relative to *Baseline*, were found during *Peak* for Eye Ratio and Mouth Position. Eye Ratio also differed from *Baseline* during *All* and *Non-Peak*. Specifically, Eye Ratio was significantly reduced in *All* compared with *Baseline* (Difference, −0.0781 AU, paired *t* test, *p* < 0.005). Eye Ratio was also significantly lower than *Baseline* in both *Peak* and *Non-Peak* epochs (*Baseline*, *Peak*, and *Non-Peak*, repeated-measures mixed-effects model, *p* = 0.0025; post hoc Tukey's correction, *Peak* vs *Baseline* median difference = −0.0742 AU; *p* < 0.05; and *Non-Peak* vs *Baseline* median difference = −0.0681 AU; *p* < 0.05). Mouth Position was also significantly lower during *Peak* versus *Baseline*, as well as during *Peak* versus *Non-Peak* (repeated-measures mixed-effects model, *p* < 0.0001; post hoc comparisons using Tukey's correction, *Peak* vs *Baseline* median difference = −2.98°; *p* < 0.005; *Peak* vs *Non-Peak* median difference = −3.49°; *p* < 0.005). All effect sizes are reported in Table 1 for CGRP facial grimace parameters. No significant differences from *Baseline* or between epochs were found in Ear Ratio, Ear Position, Ear Tip Tilt, or Snout Position. Additionally, no significant differences were observed in any measures in saline-injected mice (Extended Data Fig. 2-1).

While the absolute change of several of these measurements was small in magnitude, the observed effect size was much larger. The effect sizes for the Facial Grimace and Body Position measurements are shown in Tables 1 and 2, respectively. Here, the absolute change of the medians of *Baseline* compared with *Peak* is −2.98°, but this change has a large effect of *d* = 1.65.

**Table 1.** Facial grimace measurement effect sizes for CGRP experiments

Parameter	Comparison to Baseline	Effect size (*d*)
Ear Ratio	All	−0.23
	Peak	−0.21
	Non-Peak	−0.44
Ear Position	All	−0.57
	Peak	−1.45^†^
	Non-Peak	−0.22
Ear Tip Tilt	All	0.24^
	Peak	−0.43
	Non-Peak	−0.31
Eye Ratio	All	2.23^†^
	Peak	1.53^†^
	Non-Peak	1.74^†^
Mouth Position	All	−0.29
	Peak	1.65^†^
	Non-Peak	0.05
Snout Position	All	−0.5
	Peak	0.48
	Non-Peak	0.26

Effect sizes are reported as Cohen's *d* except in the comparison denoted with a ^. In this case the effect size is reported as a rank biserial correlation as one of the datasets was not normally distributed. Large effect sizes (>0.8) are indicated with a dagger.

**Table 2.** Body position measurement effect sizes for CGRP experiments

Parameter	Comparison to Baseline	Effect size (*d*)
Nose Tip Position	All	0.18
	Peak	0.59
	Non-Peak	0.05
Percent in Top	All	0.8
	Peak	2.19^†^
	Non-Peak	0.96^†^
Vertical Line Crosses	All	0.31
	Peak	0.99^†^
	Non-Peak	0.15
Face Inclination	All	−0.8^†^
	Peak	−1.12^†^
	Non-Peak	−0.55

Effect sizes are reported as Cohen's *d.* Large effect sizes (>0.8) are indicated with a dagger.

### Body position parameters in mice experiencing migraine after CGRP injection

A subset of the pose estimated points used for measuring facial grimace were additionally applied to generate measurements of mouse body position that could indicate pain. Body position parameters were chosen based on the observation of changed locomotive behaviors (Hasriadi et al., 2021), whereby mice were observed to explore, rear, and generally move less during pain states, as well as maintain a hunched posture more often. Body position measurements were therefore quantified to grossly incorporate changes in these complex behaviors (Fig. 3), namely, the “Relative Nose Tip Position” (Fig. 3*A*; related to exploring), “Percentage of Nose Tip in Top 1/3 of Chamber” (Fig. 3*B*; captured rearing), “Vertical Line Crosses of Nose Tip” (Fig. 3*C*; measured turning), and “Face Inclination as Slope of Nose Tip to Neck” (Fig. 3*D*; showed hunching).

**Figure 4.**
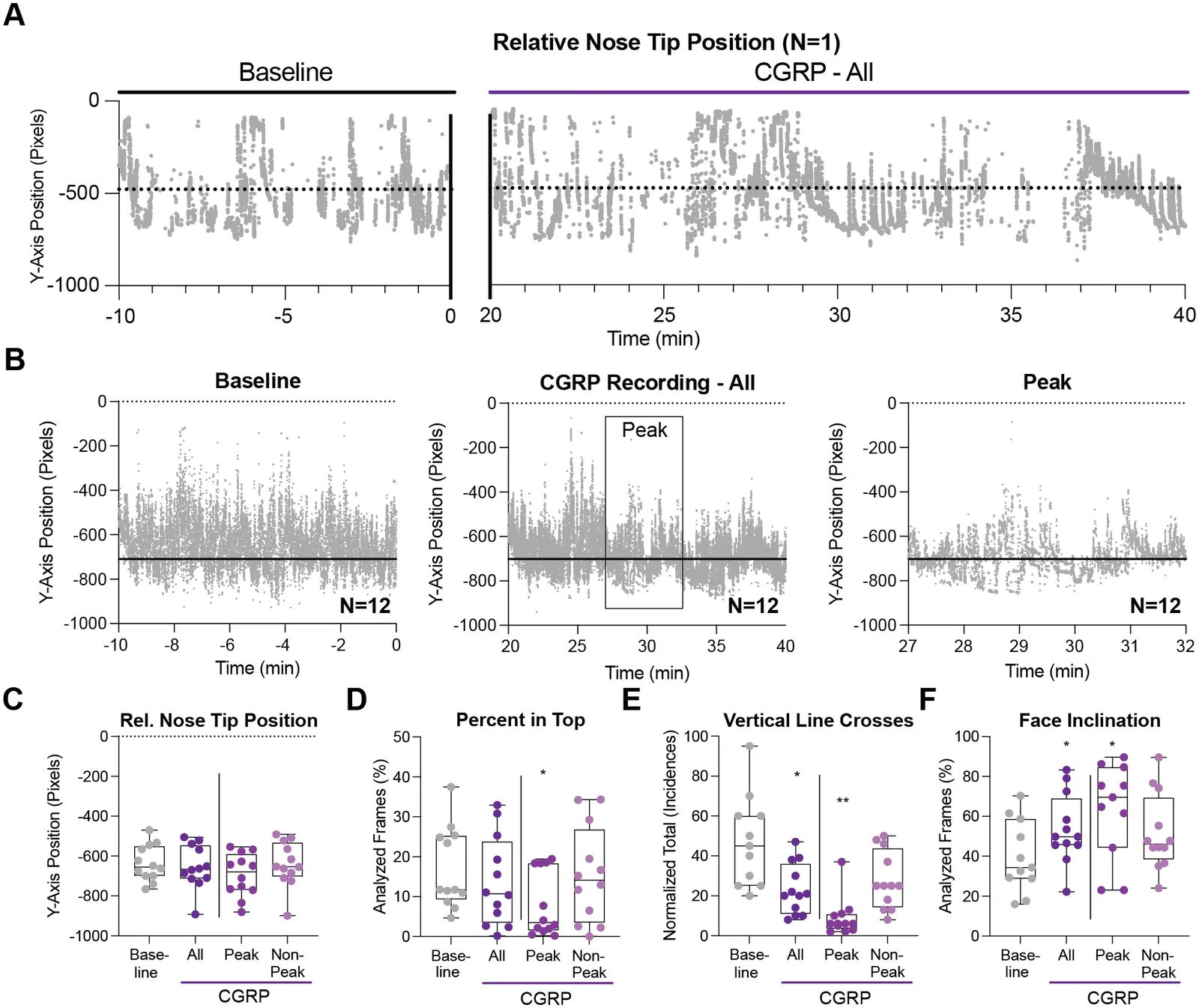
Body position parameters in mice experiencing migraine after CGRP injection. ***A***, Example of the Relative Nose Tip Position data from the baseline and CGRP time periods of one mouse (*N* = 1). Each dot is the measurement of *Y*-axis Nose Tip Position from a single frame of video. The line at *Y*-axis −450 was used to calculate the Percent in Top measurement. ***B***, Averaged data for each frame of video for Relative Nose Tip Position from 12 mice (*N* = 12) during *Baseline* and the entire CGRP Recording. The right graph shows the average Relative Nose Tip Position for 12 mice during the peak time period as indicated in Figure 2*A* and the box in the middle graph. The black horizontal line is the median of the baseline time period as a reference in all graphs. ***C–F***, The following graphs show the averaged data for each body position measurement indicated. Each dot is the averaged data for one mouse. *Baseline* is the averaged data for each mouse for all frames from the *Baseline* video. “*All*” is the averaged data for each mouse for all frames after CGRP injection. “*Peak*” is the averaged data from minutes 27–31 and after CGRP injection. “*Non-Peak*” is the averaged data from time outside of the peak time period (minutes 20–26 and minutes 32–40). Box and whiskers have a line at the median and the box is the interquartile range with the whiskers extending to the maximum and minimum value of the data. ***C***, Rel. Nose Tip Position. ***D***, Percent in Top: *Baseline* compared with *Peak* and *Non-Peak*, repeated-measures one-way ANOVA, *p* = 0.0221; post hoc comparisons using Tukey's correction; *Baseline* compared with *Non-Peak*; *p = 0.0266. ***E***, Vertical Line Crosses, The absolute number of crosses was normalized to a 5 min time period to compare baseline (10 min) and *All* (20 min) to *Peak* (5 min) and *Non-Peak* (15 min). *Baseline* compared with *All*, paired, two-tailed *t* test; *p = 0.0241; *Baseline* compared with *Peak* and *Non-Peak*, repeated-measures mixed-effects model; **p = 0.0004; post hoc comparisons using Tukey's correction; *Baseline* compared with *Peak*; ***p = 0.0025; post hoc comparisons using Tukey's correction, *Peak* compared with *Non-Peak*, ***p = 0.0002. ***F***, Face Inclination, *Baseline* compared with *All*, paired, two-tailed *t* test; *p = 0.0236; *Baseline* compared with *Peak* and *Non-Peak*, repeated-measures mixed-effects model; *p = 0.03; post hoc comparisons using Tukey's correction, *Baseline* compared with *Peak*; *p = 0.0293. If no statistics noted, data were not significantly different.

Body position parameters (Fig. 4) were plotted in the same way as facial grimace measurements. A representative diary plot shows relative nose tip position during migraine (Fig. 4*A*). Superimposed plots from all mice for Relative Nose Tip Position (shown in Fig. 4*B*) illustrate a subtle decrease in variability during *Peak*. Appropriate statistical tests were performed to compare groups, and no significant differences were found for Relative Nose Tip Position (Fig. 4*C*). In contrast, *Peak* was significantly different compared with *Baseline* for Percentage in Top (Fig. 4*D*; repeated-measures one-way ANOVA, *p* = 0.022; post hoc comparisons using Tukey's correction, *Peak* vs *Baseline* median difference = −8.25 percentage points; *p* < 0.05), for Vertical Line Crosses (Fig. 4*E*, repeated-measures mixed-effects model, *p* = 0.0004; post hoc comparisons using Tukey's correction, *Peak* vs *Baseline* median difference = −39 incidence; *p* < 0.005), and for Face Inclination (Fig. 4*F*, repeated-measures mixed-effects model, *p* = 0.03; post hoc comparisons using Tukey's correction, *Peak* vs *Baseline* median difference = 35.14 percentage points; *p* < 0.05). Reductions in *All*, compared with *Baseline* were found for both Vertical Line Crosses (paired *t* test, *All* vs *Baseline* median difference = −24.5 incidences; *p* < 0.05) and Face Inclination (paired *t* test, *All* vs *Baseline* median difference = 15.46 percentage points; *p* < 0.05). Additionally, Vertical Line Crosses was significantly decreased during *Peak* versus *Non-Peak* (paired *t* test, *Peak* vs *Non-Peak* median difference = −19 incidences; *p* < 0.001). All effect sizes are reported in Table 2 for CGRP body position parameters.

### Combined facial grimace and body position scores provide detection of pain in mice

**Figure 5.**
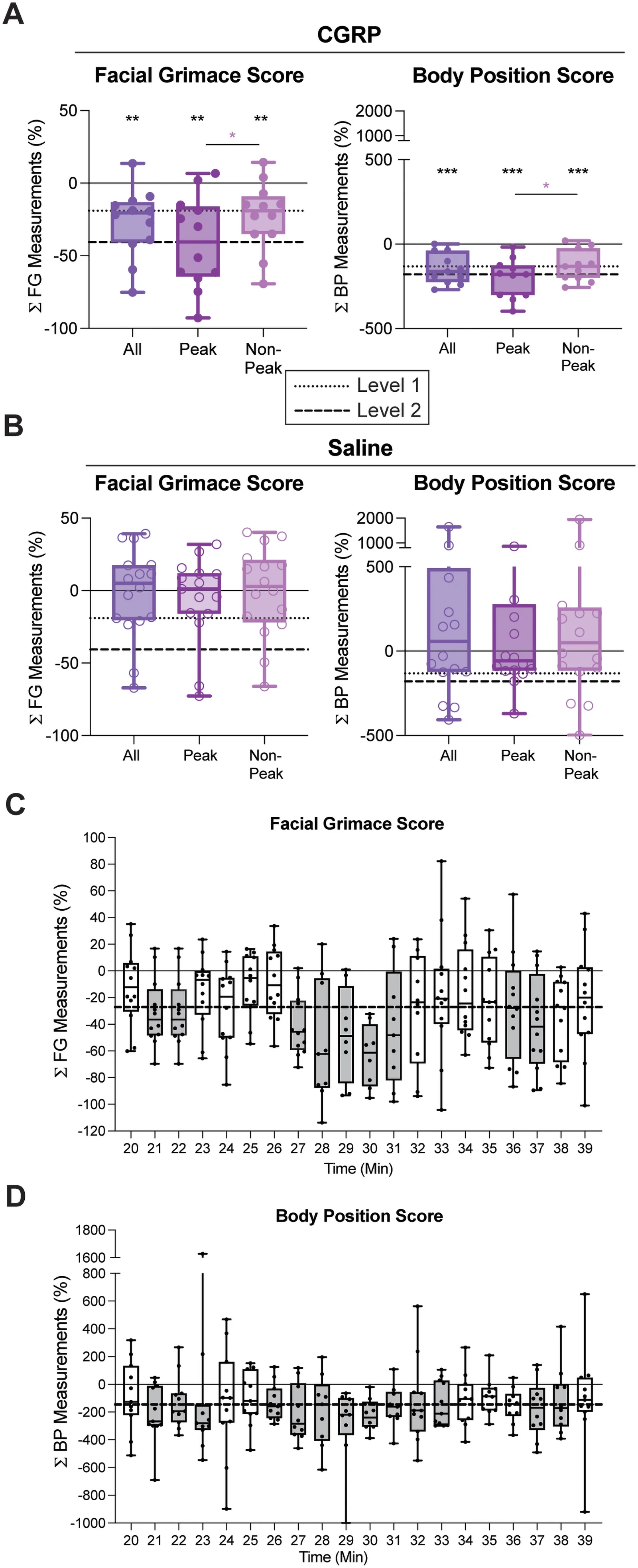
Combined Facial Grimace and Body Position Scores provide quantification of pain-related behavior in mice. Facial Grimace Scores were calculated as the added percentage change from baseline for all facial grimace measurements for each mouse. When a facial grimace parameter resulted in a positive change from baseline (Ear Ratio, Ear Position), it was inverted so the percentage change from baseline associated with pain was a negative value. Body Position Scores were calculated as the added percentage change from baseline for all position measurements for each mouse. When a body position measurement resulted in a positive change from baseline (Rel. Nose Tip Position, Face Inclination), it was inverted so the percentage change from baseline associated with pain was a negative value as with the Facial Grimace Score. ***A***, Facial Grimace (left) and Body Position (Right) Scores for mice injected with CGRP (*N* = 12). Box and whiskers have a line at the median and the box is the interquartile range with the whiskers extending to the maximum and minimum value of the data. CGRP Facial Grimace Score, *Baseline* compared with *All*, paired, two-tailed *t* test; **p = 0.0022; *Baseline* compared with *Peak*, paired, two-tailed *t* test; **p = 0.001; *Baseline* compared with *Non-Peak*, paired, two-tailed *t* test; *p = 0.0063. *Peak* compared with *Non-Peak*, paired, two-tailed, *t* test; *p = 0.0263. The dotted line is defined as the score associated with Pain Level 1 and begins at the median of the *Non-Peak* data (median = −19.04 percent change from baseline). The dashed line is defined as the score associated with Pain Level 2 and begins at the mean of the *Peak* data (median = −40.62 percent change from baseline). CGRP Body Position Score (Right); *Baseline* compared with *All*, paired, two-tailed *t* test; ***p = 0.0003; *Baseline* compared with *Peak*, paired, two-tailed *t* test, ***p = 0.002; *Baseline* compared with *Non-Peak*, paired, two-tailed *t* test; *p = 0.001. *Peak* compared with *Non-Peak*, paired, two-tailed *t* test; **p = 0.0369. Box and whiskers have a line at the median, and the box is the interquartile range with the whiskers extending to the maximum and minimum value of the data. The dotted line is defined as the score associated with Pain Level 1 and begins at the median of the *Non-Peak* data (median = −132.4 percent change from baseline). The dashed line is defined as the score associated with Pain Level 2 and begins at the mean of the Peak data (median = −179.5 percent change from baseline). For a mouse to be experiencing pain, the scores must be lower than −19.04 for Facial Grimace and −132.4 for Position. ***B***, Facial Grimace (left) and Body Position (right) Scores for mice injected with saline (*N* = 16). Box and whiskers have a line at the median, and the box is the interquartile range with the whiskers extending to the maximum and minimum value of the data. Statistical tests were not significant. Saline Facial Grimace Score, *Baseline* compared with *All*, paired, two-tailed *t* test, *p* = 0.8211. *Baseline* compared with *Peak* and *Non-Peak*, repeated-measures mixed model, *p* = 0.5096. Saline Body Position Score: *Baseline* compared with *All*, paired, two-tailed *t* test, *p* = 0.1673. *Baseline* compared with *Peak* and *Non-Peak*, repeated-measures mixed model, *p* = 0.2735. ***C***, The percentage change from baseline for each facial grimace measurement was added together and averaged for each minute of the CGRP recording. Each dot corresponds to one animal. The dashed line is the median of all 20 min for all mice (−30.12% change from baseline). Minutes where the median falls below the dashed line are colored gray. Minutes 27–31 show the longest string of median scores below the dashed line. ***D***, The percentage change from baseline for each body position measurement was added together and averaged for each minute of the CGRP recording. Each dot corresponds to one animal. The dashed line is the median of all 20 min for all mice (−174.41% change from baseline). Minutes where the median falls below the dashed line are colored gray. Minutes 27–34 have the longest string of lowest median scores. For extended data showing the percentage change from baseline used to calculate the Facial Grimace and Body Position Scores, see Extended Data Figure 5-1.

Individual facial grimace and body position parameters were combined to create two separate composite scores. Each measurement was first converted to a percentage change from *Baseline* (Extended Data Fig. 5-1), and these were then added together to calculate the summed values for facial grimace or for body position for both CGRP- and saline-injected mice (Fig. 5). CGRP-injected mice showed significant changes between *Baseline* and *All* (referring to all frames recorded) in the summed measurements for both Facial Grimace and Body Positions Scores, while saline-injected mice showed no changes (Fig. 5*A*,*B*). Pain-related behavior was defined as a significant difference from Baseline in both Facial Grimace and Body Position Scores. Specifically, CGRP significantly lowered both Facial Grimace Score and Body Position Score in *All* relative to *Baseline* (Facial Grimace Score, paired *t* tests, *All* vs *Baseline* median difference = −20.59 percentage points; *p* < 0.005; effect size, *d* = −1.14; Body Position Score, paired *t* tests, *All* vs *Baseline* median difference = −162.6 percentage points; *p* < 0.001; effect size, *d* = −1.6). These data show that pain-related behavior can be detected in both the composite scores of facial grimace and body position using this approach.

10.1523/ENEURO.0064-26.2026.f5-1 Figure 5-1 Percentage Change from Baseline for each measurement used to generate Facial Grimace and Body Position Scores for Saline- and CGRP-injected mice **A**) Graphs of Facial Grimace (left) and Body Position (right) parameters for CGRP-injected mice (N = 12). Each dot is the average of one mouse for the indicated time-period after being transformed into a percentage compared to the baseline time-period. If a measurement resulted in a positive change from baseline (Ear Ratio, Ear Position, Rel. Nose Tip Position, Face Inclination), it was inverted so the percentage change from baseline associated with pain was a negative value. Box and whiskers have a line at the median and the box is the interquartile range with the whiskers extending to the maximum and minimum value of the data. Eye Ratio: *Baseline* compared to *All*, paired, two-tailed t-test, p = 0.0002; *Baseline* compared to *Peak* and *Non-Peak*, repeated measured mixed model, p = 0.0018, ; post hoc comparisons using Tukey’s correction, *Baseline* compared to *Peak*, *, p = 0.0105, *Baseline* compared to *Non-Peak*, **, p = 0.0024. Mouth Position: *Baseline* compared to *Peak* and *Non-Peak*, repeated measured mixed model, p = 0.008 ; post hoc comparisons using Tukey’s correction, Baseline compared to *Peak*, *, p = 0.0355, *Peak* compared to *Non-Peak*, *, p = 0.0417. Percent in Top: *Baseline* compared to *Peak* and *Non-Peak*, repeated measures Friedman Test, p = 0.0435 ; post hoc comparisons using Dunn’s correction, *Baseline* compared to *Peak*, *, p = 0.0315. Vertical Line Crosses: *Baseline* compared to *Peak* and *Non-Peak*, repeated measured mixed model, ns, p = 0.6286 ; post hoc comparisons using Tukey’s correction, *Baseline* compared to *Non-Peak*, *, p = 0.019. Face Inclination: *Baseline* compared to *All*, paired two-tailed t-test, *, p = 0.0255. If no statistics noted, data was not significantly different. **B**) Graphs of Facial Grimace (left) and Body Position (right) parameters for saline-injected mice (N = 16). Each open circle is the average of one mouse for the indicated time-period after being transformed into a percentage compared to the baseline time-period. If a measurement resulted in a positive change from baseline (Ear Ratio, Ear Position, Rel. Nose Tip Position, Face Inclination), it was inverted so the percentage change from baseline associated with pain was a negative value. Box and whiskers have a line at the median and the box is the interquartile range with the whiskers extending to the maximum and minimum value of the data. Eye Ratio: *Baseline* compared to *Peak* and *Non-Peak*, repeated measures mixed model, p = 0.0381. Vertical Line Crosses: *Baseline* compared to *All*, Wilcoxon signed-rank test, p = 0.0494; *Baseline* compared to *Peak* and *Non-Peak*, repeated measures one-way ANOVA, p = 0.0379. If no statistics noted, data was not significantly different. All groups were tested for normality. In cases where the data from all groups was not normally distributed, a Wilcoxon Ranked sum test was used for comparisons of *Baseline* to *All* and a Friedman test was used for the comparison of *Baseline* to *Peak* and *Non-Peak*. Download Figure 5-1, TIF file.

### Defining two pain-related behavior levels based on thresholds calculated during distinct time windows after inducing migraine

Migraine induced by the injection of CGRP has been reported to cause a peak in the pain intensity in mice at ∼30 min postinjection (Rea et al., 2018). While prior studies have demonstrated this peak using a lower sampling rate (e.g., four images per time point, acquired 20 s apart), the current approach uses a higher sampling rate and therefore provides a more detailed assessment of the timeline of behavioral changes after inducing migraine in mice. Using this new approach, the empirical *Peak* of migraine pain-related behavior emerged around the expected peak from *t* = 27–32 min postinjection of CGRP, further defined below (Fig. 5*C*,*D*). The remaining time, both before and after the defined *Peak* time period, was combined to calculate the *Non-Peak* period. The composite scores were compared with *Baseline* for both *Peak* and *Non-Peak* periods (Fig. 5*A*,*B*), where both *Peak* and *Non-Peak* differed significantly from *Baseline* (*Peak* vs *Baseline* median difference = −40.62 percentage points; *p* = 0.001; effect size, *d* = −1.28; and *Non-Peak* vs *Baseline* median difference = −19.04 percentage points; *p* < 0.01; effect size, *d* = −0.97; Body Position Score, *Peak* vs *Baseline* median difference = −179.5 percentage points; *p* < 0.001; effect size, *d* = −1.76; and *Non-Peak* vs *Baseline* median difference = −132.4 percentage points; *p* = 0.001; effect size, *d* = −0.97). *Peak* also differed significantly from *Non-Peak* in CGRP-injected mice for both Facial Grimace Score (paired *t* test, *Peak* vs *Non-Peak* median difference = −21.58 percentage points; *p* < 0.05; effect size, *d* = 0.74) and Body Position Score (paired *t* test, *Peak* vs *Non-Peak* median difference = −47.1 percentage points; *p* < 0.05; effect size, *d* = 0.73). These data indicate that mouse pain-related behavior during the empirical *Peak* is distinct from that during *Non-Peak* periods. The *Non-Peak* and *Peak* median values were therefore used to create thresholds for two levels of pain-related behavior, as indicated in Figure 5, *A* and *B*: Level 1 (dotted line; −19.04 percentage points difference from *Baseline* for facial grimace and −132.4 percentage points for body position) and Level 2 (dashed line; −40.62 percentage points difference from *Baseline* for facial grimace and −179.5 percentage points for body position).

Selection of the empirical peak which led to the use of *Peak* and *Non-Peak* periods throughout this study (starting with Fig. 2) was based on diary plots binned into one-minute segments across the entire CGRP recording period (Fig. 5*C*,*D*). Scores for each 1 min segment were the summed value of all individual measurements as a median percent difference from *Baseline*. It was found that from minute 27 to 32, the binned scores were below the mean value of *All* CGRP frames in Facial Grimace Score (−27.17 percentage points, Fig. 5*C*, gray bars) and from minute 26 to 33, they were below the mean value of *All* CGRP frames in Body Position Scores (−145.7 percentage points, Fig. 5*D*, gray bars). The empirical *Peak* was therefore selected based on the criteria where both scores were below the mean of *All* CGRP frames for both scores. It is noteworthy that other 1 min binned values outside of the empirical peak fall below the mean from the *All* CGRP recording (Fig. 5*C*,*D*, gray bars); however, the longest continuous period where both scores meet these criteria was used as the empirical *Peak*. This method confirmed the previously reported occurrence of the peak of CGRP-induced migraine pain ∼30 min after injection (Rea et al., 2018). Due to a higher sampling rate and continuous recording in the present study (30 frames/s, 20 min recording time), the empirical *Peak* was found to span 5 min and was used to define a second pain-related behavior level.

In summary, automated analysis of video recordings of mouse behavior using both Facial Grimace and Body Position Scores provides a high-throughput approach to quantify persistent pain-related behavior in mice. This method was built on established methods for detecting facial grimace (Le Moëne and Larsson, 2023) and combined this with body position changes using a single camera in the same chamber. Validation of this method using CGRP-induced migraine demonstrated the ability to detect persistent pain-related behavior and further allowed for the defining of two migraine pain-related behavioral thresholds (Levels 1 and 2).

### Behavioral detection of sound-evoked pain-related behavior

Having validated the above method to measure persistent pain-related behavior from video recordings of mouse behavior, this approach was next used to detect changes in behavior elicited by prolonged sound exposure. Sensitivity to sound in mice has been detected primarily using behavior measurements in response to sound onset (Hickox and Liberman, 2014; Salloum et al., 2016; Manohar et al., 2020), which is an effective way to monitor changes in sound sensitivity. To measure behavioral changes associated with pain caused by longer duration sound, the following experiments used facial grimace and body position measurements to quantify sound-evoked pain and determine the level of sound that caused pain-related behaviors in mice.

### Sound exposure elicits changes in individual measurements of facial grimace and body position

**Figure 6.**
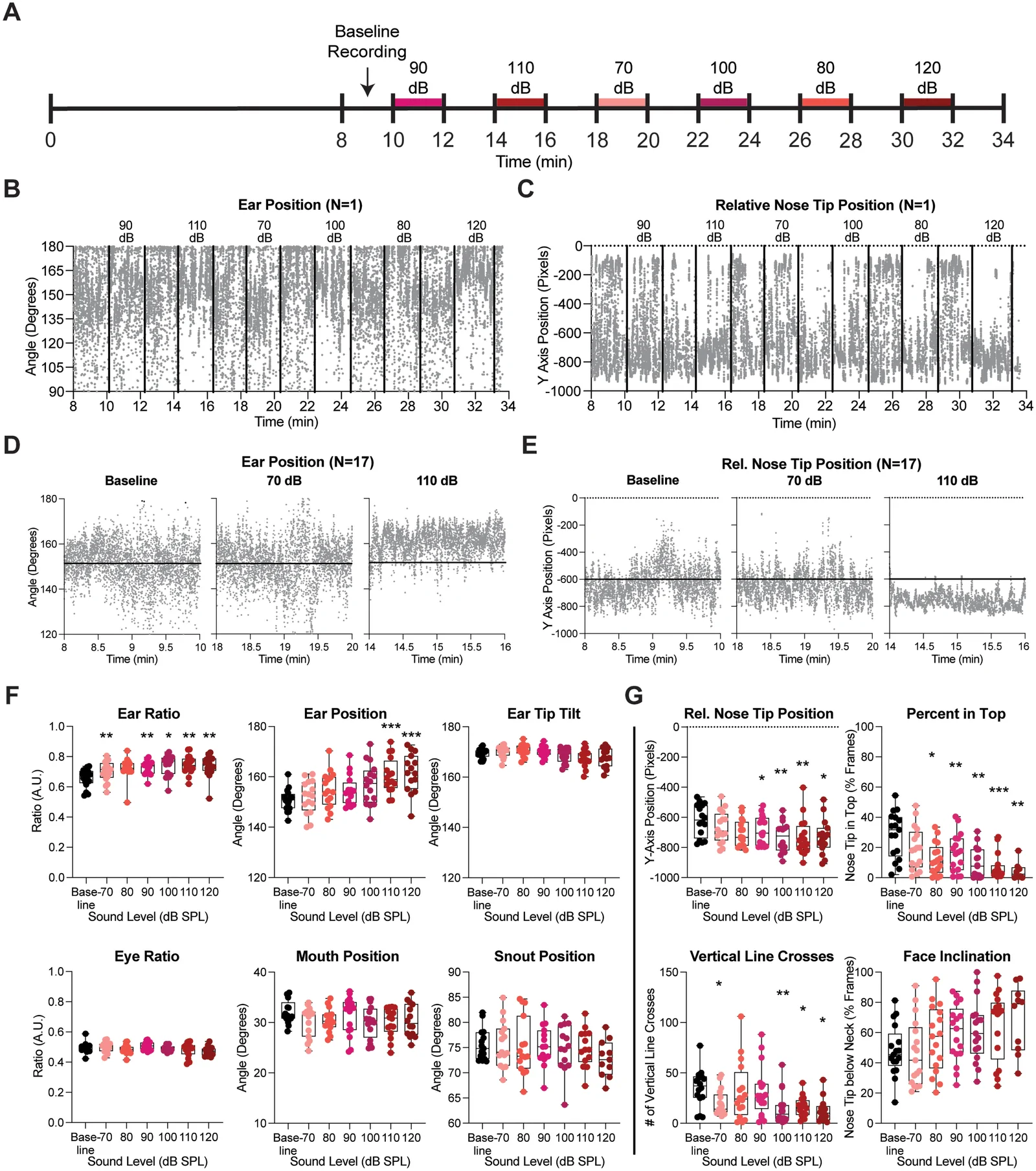
Sound exposure elicits changes in individual measurements of facial grimace and body position. ***A***, Timeline of interleaved sound level experiments. Recording started at 8 min after the mice were placed in the acoustic chamber. The recording from 8 to 10 min was used as the baseline recording. Sound exposure was initiated in 2 min time periods separated by 2 min of silence. Sound was presented in out of the decibel-level order as indicated by color and label. ***B***, Example of analyzed frames of Ear Position measurements for one mouse for the entirety of one recording (*N* = 1). The black vertical lines indicate when the sound levels were manually changed on or off. ***C***, Example of analyzed frames of Relative Nose Tip Position measurements for one mouse for the entirety of one recording (*N* = 1). The black vertical lines indicate when the sound levels were manually changed on or off. ***D***, Averaged Ear Position measurements of analyzed frames from 12 mice (*N* = 12) for *Baseline*, 70 dB, and 110 dB time periods. The time in minutes is the average on and off time periods for *Baseline* and 70 and 110 dB for each recording. The 70 dB recording occurred after the 110 dB recording, but the graphs have been reordered according to the decibel level. Horizontal black lines indicate the average for the baseline graph. ***E***, Averaged Relative Nose Tip Position measurements of analyzed frames from 12 mice (*N* = 12) for *Baseline* and 70 and 110 dB time periods. The time in minutes is the average on and off time periods for *Baseline*, 70 and 110 dB for each recording. The 70 dB recording occurred after the 110 dB recording, but the graphs have been reordered according to decibel level. Horizontal black lines indicate the average for the baseline graph. ***F***, The following graphs show the averaged data for each facial parameter indicated. Each dot is the averaged data for the indicated 2 min time period for one mouse. The sound levels have been reordered from the lowest to the most intense sound level. Box and whiskers have a line at the median, and the box is the interquartile range with the whiskers extending to the maximum and minimum value of the data. Ear Ratio, baseline compared with each sound level, repeated-measures mixed model, *p* = 0.0013; post hoc comparison using Tukey's correction, baseline compared with 70 dB,**p = 0.0068; baseline compared with 90 dB; **p = 0.0055; baseline compared with 100 dB; *p = 0.0135; baseline compared with 110 dB; **p = 0.0079; baseline compared with 120 dB; **p = 0.0015. Ear Position, baseline compared with each sound level, repeated-measures mixed model; *p* = <0.0001; post hoc comparison using Tukey's correction, baseline compared with 110 dB; ***p = 0.0001; baseline compared with 120 dB; ***p = 0.0008. Ear Tip Tilt, baseline compared with each sound level; repeated-measures mixed model; *p* = 0.0148; Eye Ratio, baseline compared with each sound level; repeated-measures mixed model; *p* = 0.0354; Mouth Position, baseline compared with each sound level; repeated-measures mixed model; ns, *p* = 0.1308. Snout Position, baseline compared with each sound level; repeated-measures mixed model; ns, *p* = 0.5743. ***G***, The following graphs show the averaged data for each body position parameter indicated by the title. Each dot is the averaged data for the indicated 2 min time period for one mouse. The sound levels have been reordered from the lowest to the most intense sound level. Box and whiskers have a line at the median, and the box is the interquartile range with the whiskers extending to the maximum and minimum value of the data. Rel. Nose Tip Position, baseline compared with each sound level; repeated-measures mixed model; *p* = 0.0016; post hoc comparison using Tukey's correction; baseline compared with 90 dB; *p = 0.0142; baseline compared with 100 dB; **p = 0.0095; baseline compared with 110 dB; **p = 0.0058; baseline compared with 120 dB; *p = 0.0473. Percent in Top, baseline compared with each sound level; repeated-measures mixed model; *p* = <0.0001; post hoc comparison using Tukey's correction; baseline compared with 80 dB; *p = 0.0186; baseline compared with 90 dB; **p = 0.0022; baseline compared with 100 dB; **p = 0.0026; baseline compared with 110 dB; ***p = 0.0005; baseline compared with 120 dB; **p = 0.0066. Vertical Line Crosses, baseline compared with each sound level; repeated-measures mixed model; *p* = 0.0078; post hoc comparison using Tukey's correction; baseline compared with 70 dB; *p = 0.0112; baseline compared with 100 dB; **p = 0.0018; baseline compared with 110 dB; *p = 0.0122; baseline compared with 120 dB; *p = 0.0122. Face Inclination, baseline compared with each sound level, repeated-measures mixed model; *p* = 0.0221.

Mouse behavior was next tested during a sound exposure protocol with varying sound intensities using an assessment of facial grimace and body position parameters as described above. Individual facial grimace and body position parameters changed with increasing sound intensities (Fig. 6). The experimental design is shown in Figure 6*A*. Video recordings started 8 min after the mice were placed in the acoustic chamber. The recording from 8 to 10 min was used as the baseline recording. Sound exposures of varied sound intensity levels in a set sequence were performed in 2 min time periods and were interleaved with 2 min time periods of no sound exposure (“*No Sound*”). The highest sound exposure level (120 dB) was placed last in the sequence of exposures, assuming that lasting cochlear damage could occur.

Example diary plots of one mouse show individual measures of Ear Position and Relative Nose Tip Position in response to the sound exposure protocol (Fig. 6*B*,*C*). Superimposed data from all mice (*n* = 17) visualize the results from *Baseline*, 70 dB SPL, and 110 dB SPL periods (Fig. 6*D*,*E*; raw and labeled video examples shown in Movies 2 and 3). Median values along with appropriate statistical tests revealed differences compared with *Baseline* in multiple individual measurements of facial grimace and body position at many intensities of sound. Ear Ratio, Ear Position, Relative Nose Tip Position, Percent in Top, and Vertical Line Crosses were all significantly different at 110 dB SPL compared with *Baseline* (Fig. 6*F*,*G*). Specifically, Ear Ratio (repeated-measures mixed-effects model of all groups, *p* = 0.0013; post hoc comparisons using Tukey's correction; 70 dB vs *Baseline* median difference = 0.0361 AU; *p* < 0.01; effect size, *d* = 0.7; 90 dB vs *Baseline* median difference = 0.0602 AU; *p* < 0.01; effect size, *d* = 1.27; 100 dB vs *Baseline* median difference = 0.0927 AU; *p* < 0.05; effect size, *d* = 1.35; 110 dB vs *Baseline* median difference = 0.0637 AU; *p* < 0.01; effect size, *d* = 1.46; and 120 dB vs *Baseline* median difference = 0.0642 AU; *p* < 0.01; effect size, *d* = 1.26), Ear Position (repeated-measures mixed-effects model of all groups, *p* < 0.0001; post hoc comparisons using Tukey's correction, 110 dB vs *Baseline* median difference = 8.0°; *p* < 0.001; effect size, *d* = 1.51; 120 dB vs *Baseline* mean difference = 10.6°; *p* < 0.001; effect size, *d* = 1.74; Table 3), Relative Nose Tip Position (repeated-measures mixed-effects model of all groups, *p* = 0.0016; post hoc comparisons using Tukey's correction; 90 dB vs *Baseline* median difference = −87 pixels; *p* < 0.05; effect size, *d* = −0.66; 100 dB vs *Baseline* median difference = −106 pixels; *p* < 0.01; effect size, *d* = −1.07; 110 dB vs *Baseline* median difference = −148 pixel; *p* < 0.01; effect size, *d* = −0.96; 120 dB vs *Baseline* median difference = −114 pixels; *p* < 0.05; effect size, *d* = −1.06; Table 4), Percent in Top (repeated-measures mixed-effects model of all groups, *p* < 0.0001; post hoc comparisons using Tukey's correction; 80 dB vs *Baseline* median difference = −21.4 percentage points; *p* < 0.05; effect size, *d* = −1.21; 90 dB vs *Baseline* median difference = −15.0 percentage points; *p* < 0.01; effect size, *d* = −0.84; 100 dB vs *Baseline* median difference = −24.1 percentage points; *p* < 0.01; effect size, *d* = −1.44; 110 dB vs *Baseline* median difference = −28.9 percentage points; *p* < 0.001; effect size, d = −1.74; 120 dB vs *Baseline* median difference = −29.4 percentage points; *p* < 0.01; effect size, *d* = 1.79), and Vertical Line Crosses (repeated-measures mixed-effects model of all groups, *p* = 0.0078; post hoc comparisons using Tukey's correction; 70 dB vs *Baseline* median difference = −23 incidences; *p* < 0.05; 100 dB vs *Baseline* median difference = −28 incidences; *p* < 0.01; effect size, *d* = −0.94; 110 dB vs *Baseline* median difference = −22 incidences; *p* < 0.05; effect size, *d* = −1.26; 120 dB vs *Baseline* median difference = −27 incidences; *p* < 0.05; effect size, *d* = −1.44) all showed significant differences at multiple intensities. Ear Tip Tilt, Eye Ratio, and Face Inclination were all significant in the mixed-effects model but showed no significant post hoc comparisons. Specifically, Ear Tip Tilt (repeated-measures mixed-effects model of all groups, *p* **=** 0.0148), Eye Ratio (repeated-measures mixed-effects model of all groups, *p* = 0.0354), and Face Inclination (repeated-measures mixed-effects model of all groups, *p* = 0.0221) showed significant group effects. Finally, Mouth and Snout Position measures had no significant group effects. While the changes of each measurement may not reach significance, the effect size for many comparisons was larger than the change in magnitude of the measurement may suggest. The effect sizes for each facial grimace and body position measurement per sound level are presented in Tables 3 and 4. In the case of Mouth Position where the changes were not significant, the effect sizes are medium (between 0.5 and 0.8). Taken together, these data suggest that sound-evoked pain-related behavior may be preferentially detected by individual grimace measurements related to ears and by changes in body position.

**Movie 2.** Interleaved sound experiment example. Ten-second video clips from the original recording of the *Baseline*, 110 dB SPL, post 110 dB SPL, and 70 dB SPL of a mouse undergoing the interleaved sound protocol. Sound is included in the video. [View online]

**Movie 3.** Labeled, interleaved sound experiment example. Ten-second video clips from the labeled recording of the *Baseline*, 110 dB SPL, post 110 dB SPL, and 70 dB SPL time periods of a mouse undergoing the interleaved sound protocol. The labeled video is from the same time periods from the same mouse shown in Movie 2. The labels were generated by DLC, and any marker above 0.6 likelihood is shown for visualization. Only markers with a likelihood above 0.95 were used for analysis. [View online]

**Table 3.** Facial grimace measurement effect sizes for sound assay experiments

Parameter	Comparison	Effect size (*d*)
Ear Ratio	Baseline versus 70 dB	−0.7
	Baseline versus 80 dB	−1.13^†^
	Baseline versus 90 dB	−1.27^†^
	Baseline versus 100 dB	−1.35^†^
	Baseline versus 110 dB	−1.46^†^
	Baseline versus 120 dB	−1.26^†^
Ear Position	Baseline versus 70 dB	−0.2
	Baseline versus 80 dB	−0.71
	Baseline versus 90 dB	−0.73
	Baseline versus 100 dB	−0.98^†^
	Baseline versus 110 dB	−1.51^†^
	Baseline versus 120 dB	−1.74^†^
Ear Tip Tilt	Baseline versus 70 dB	−0.27
	Baseline versus 80 dB	−0.59
	Baseline versus 90 dB	−0.23
	Baseline versus 100 dB	0.36
	Baseline versus 110 dB	0.73
	Baseline versus 120 dB	0.5
Eye Ratio	Baseline versus 70 dB	−0.14
	Baseline versus 80 dB	0.29
	Baseline versus 90 dB	−0.41
	Baseline versus 100 dB	0.28
	Baseline versus 110 dB	0.37
	Baseline versus 120 dB	0.94^†^
Mouth Position	Baseline versus 70 dB	0.7
	Baseline versus 80 dB	0.65
	Baseline versus 90 dB	0.17
	Baseline versus 100 dB	0.7
	Baseline versus 110 dB	0.77
	Baseline versus 120 dB	0.56
Snout Position	Baseline versus 70 dB	−0.02
	Baseline versus 80 dB	0.08
	Baseline versus 90 dB	0.08
	Baseline versus 100 dB	0.16
	Baseline versus 110 dB	0.17
	Baseline versus 120 dB	0.49

Effect sizes are reported as Cohen's *d.* Large effect sizes (>0.8) are indicated with a dagger.

**Table 4.** Body position measurement effect sizes for sound assay experiments

Parameter	Comparison	Effect size (*d*)
Nose Tip Position	Baseline versus 70 dB	0.43
	Baseline versus 80 dB	0.87^†^
	Baseline versus 90 dB	0.66
	Baseline versus 100 dB	1.07^†^
	Baseline versus 110 dB	0.96^†^
	Baseline versus 120 dB	1.06^†^
Percent in Top	Baseline versus 70 dB	0.61
	Baseline versus 80 dB	1.21^†^
	Baseline versus 90 dB	0.84^†^
	Baseline versus 100 dB	1.44^†^
	Baseline versus 110 dB	1.74^†^
	Baseline versus 120 dB	1.79^†^
Vertical Line Crosses	Baseline versus 70 dB	0.94^†^
	Baseline versus 80 dB	0.14
	Baseline versus 90 dB	0.17
	Baseline versus 100 dB	1.15^†^
	Baseline versus 110 dB	1.26^†^
	Baseline versus 120 dB	1.44^†^
Face Inclination	Baseline versus 70 dB	0.08
	Baseline versus 80 dB	−0.39
	Baseline versus 90 dB	−0.64
	Baseline versus 100 dB	−0.71
	Baseline versus 110 dB	−0.86^†^
	Baseline versus 120 dB	−1.13^†^

Effect sizes are reported as Cohen's *d.* Large effect sizes (>0.8) are indicated with a dagger.

### After individual sound exposures, mouse behavior returned to baseline during *No Sound* periods except after exposure to sound at 120 dB SPL

**Figure 7.**
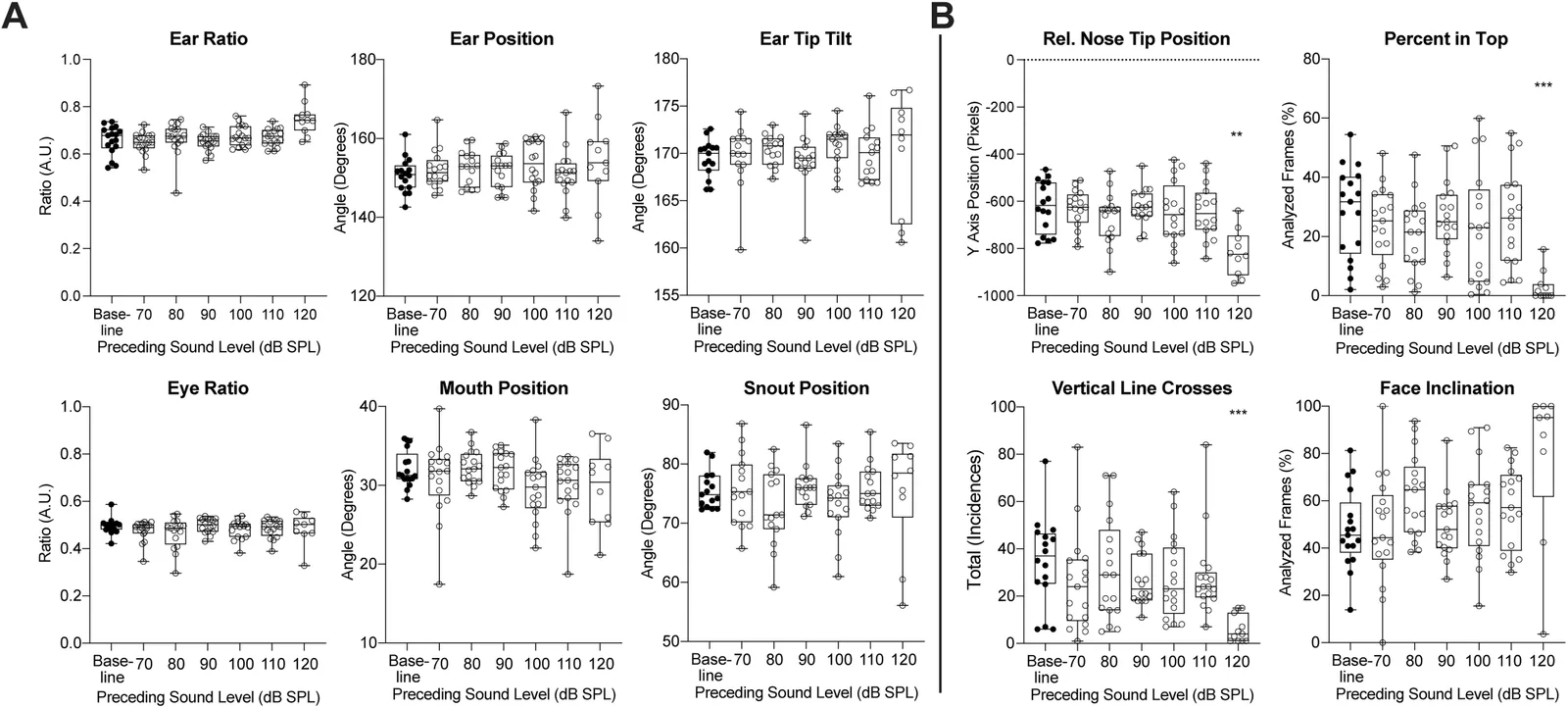
After sound exposures, mouse behavior returned to baseline during *No Sound* periods except after exposure to sound at 120 dB SPL. ***A***, The graphs show the averaged data for each facial grimace parameter indicated by the title. Each dot is the averaged data for the indicated 2 min time period for one mouse. The data have been reordered based on the preceding sound level of the no sound period from the lowest to the most intense sound level. Box and whiskers have a line at the median and the box is the interquartile range with the whiskers extending to the maximum and minimum value of the data. Ear Ratio, baseline compared with each sound level, repeated-measures mixed model, *p* = 0.0012. All other facial parameters showed no significant changes. ***B***, The graphs show the averaged data for each body position parameter indicated by the title. Each dot is the averaged data for the indicated 2 min time period for one mouse. The data have been reordered based on the preceding sound level of the silent sound period from the lowest to the most intense sound level. Box and whiskers have a line at the median, and the box is the interquartile range with the whiskers extending to the maximum and minimum value of the data. Rel. Nose Tip Position, baseline compared with each sound level, repeated-measures mixed model, *p* = 0.0003; post hoc comparison using Tukey's correction, baseline compared with 120 dB; **p = 0.002. Percent in Top, baseline compared with each sound level; repeated-measures mixed model; *p* = 0.0013; post hoc comparison using Tukey's correction; baseline compared with 120 dB; ***p = 0.001. Vertical Line Crosses, baseline compared with each sound level; repeated-measures mixed model; *p* = 0.005; post hoc comparison using Tukey's correction; baseline compared with 120 dB; ***p = 0.0001. Face Inclination, baseline compared with each sound level; repeated-measures mixed model; *p* = 0.0161.

The sound exposure protocol for individual mice included a sequence of multiple sound presentations with varying sound pressure levels, and the analysis of the resulting data, as presented in Figure 6, relies on the assumption that mouse behavior returns to baseline following each sound presentation. To verify this assumption, *No Sound* periods following each sound presentation were analyzed. In Figure 7, *No Sound* periods are arranged in an increasing order of the preceding sound exposure level, and not in the order as they were presented in the interleaved sound exposure protocol. Ear ratio showed a significant effect of group (Fig. 7*A*; repeated-measures mixed-effects model, *p* = 0.0012); however, no significant differences were detected in post hoc comparisons to *Baseline* in any *No Sound* period. The remaining facial grimace parameters showed no significant changes following any sound intensity presented compared with *Baseline*. In contrast, for most body position measurements, the *No Sound* period after 120 dB SPL sound exposure did show significant differences compared with *Baseline* (Fig. 7*B*). Specifically, Relative Nose Tip Position (repeated-measures mixed-effects model, *p* = 0.0003, post hoc comparisons using Tukey's correction, post-120 dB vs *Baseline* median difference = −207 pixels, *p* < 0.01), Percent in Top (repeated-measures mixed-effects model, *p* = 0.0013, post hoc comparisons using Tukey's correction, post-120 dB vs *Baseline* median difference = −32 percentage points; *p* < 0.001), and Vertical Line Crosses (repeated-measures mixed-effects model, *p* = 0.01; post hoc comparisons using Tukey's correction; post-120 dB vs *Baseline* median difference = −33 incidences; *p* < 0.001) all showed significant changes during *No Sound* following 120 dB SPL, while no significant differences were found in post hoc comparisons for Face Inclination (repeated-measures mixed-effects model, *p* = 0.0161). In this current study, 120 dB SPL was presented last with the consideration that this level of sound might impact subsequent animal behavior, which was confirmed here for multiple individual body position measurements.

In summary, these findings indicate that for the specific design of the sound protocol used here, after sound exposures mouse behavior returned to baseline during *No Sound* periods, except after exposure to sound at 120 dB SPL.

### Sound exposure above 100 dB SPL caused changes in behavior consistent with pain-related behavior in mice

**Figure 8.**
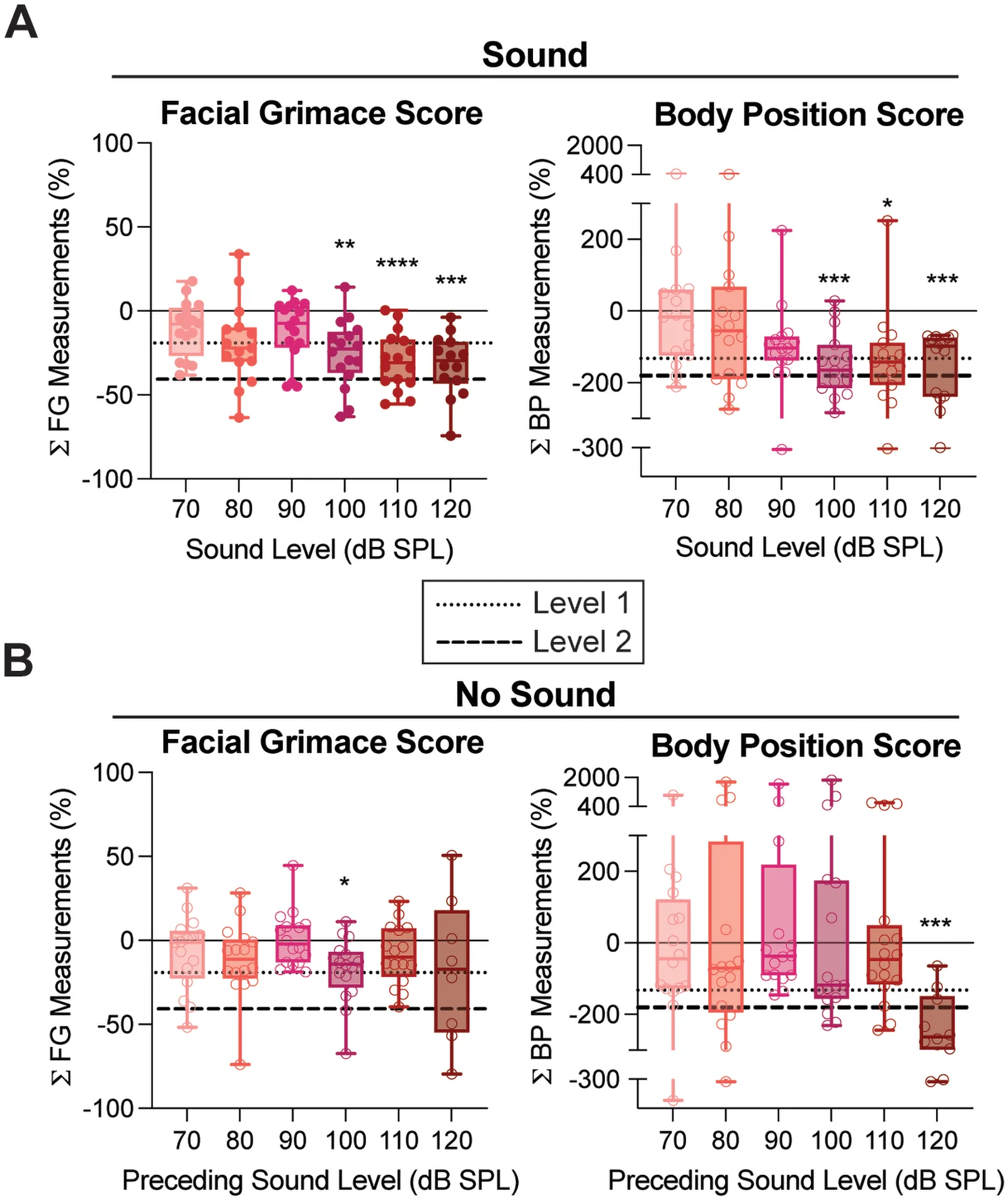
Sound exposure above 100 dB SPL caused changes in behavior consistent with pain in mice. ***A***, Facial Grimace (left) and Body Position (right) Scores for mice during exposure to sound (*N* = 17). Facial Grimace Score; Sound, repeated-measures mixed-effects model; *p* = <0.0001; post hoc comparisons using Tukey's correction; baseline compared with 100 dB; **p = 0.0025; baseline compared with 110 dB; ****p = <0.0001; baseline compared with 120 dB; ***p = 0.0003. Body Position Score; Sound, repeated-measures mixed-effects model, *p* = 0.0012; post hoc comparisons using Tukey's correction; baseline compared with 100 dB; ***p = 0.0003; baseline compared with 110 dB; *p = 0.0174; baseline compared with 120 dB; ***p = 0.0006. The Pain Level 1 threshold is −19.04 for Facial Grimace and −132.4 for Body Position (dotted lines). The Pain Level 2 threshold is −40.62 for Facial Grimace and −179.5 for Body Position (dashed lines). ***B***, Facial Grimace (left) and Body Position (right) Scores for mice during the no sound periods (*N* = 17). Box and whiskers have a line at the median and the box is the interquartile range with the whiskers extending to the maximum and minimum value of the data. Facial Grimace Score, No Sound, baseline compared with each sound level, repeated-measures mixed model; ns, *p* = 0.1157. Body Position Score, No Sound, baseline compared with each sound level, repeated-measures mixed model; ns, *p* = 0.2341. For extended data showing percentage change from baseline used to calculate Facial Grimace and Body Position scores, see Extended Data Figure 8-1.

As presented for the migraine model (Fig. 5), Facial Grimace and Body Position Scores for both *Sound* and *No Sound* were calculated as a sum of the changes from *Baseline* in individual measurements (Extended Data Fig. 8-1, Fig. 8). For comparison, thresholds for two migraine pain levels, Level 1 and Level 2, as established in the migraine model (Fig. 5), were added to Figure 8. Both composite scores for “Sound” were significantly different from *Baseline* for sound presentation at 100 dB SPL and above, thus meeting our definition of pain-related behavior. Additionally, median values of both composite scores at 100 dB SPL and above were found to surpass the migraine pain-related behavior Level 1 threshold (Fig. 8*A*; dotted line). This validates that both stimuli, sound and migraine, elicit significant behavioral changes from *Baseline*, surpassing a threshold for pain-related behavior to a similar extent. Specifically, significant differences were found for Grimace Scores (repeated-measures mixed-effects model, *p* < 0.0001; post hoc comparisons using Tukey's correction; 100 dB vs *Baseline* median difference = −22.6 percentage points; *p* < 0.01; effect size, *d* = 1.24; 110 dB vs *Baseline* median difference = −30.9 percentage points; *p* < 0.0001; effect size, *d* = 1.75; and 120 dB vs *Baseline* median difference = −29.6 percentage points; *p* < 0.001; effect size, *d* = 2.51) and Position Scores (repeated-measures mixed-effects model, *p* = 0.0012; post hoc comparisons using Tukey's correction; 100 dB vs *Baseline* median difference = −165.1 percentage points; *p* < 0.001; effect size, *d* = 2.34; 110 dB vs *Baseline* median difference = −141.9 percentage points; *p* < 0.05; effect size, *d* = 1.49; 120 dB vs *Baseline* median difference = −98.6 percentage points; *p* < 0.001; effect size, *d* = 2.35) at and above 100 dB SPL. These findings indicated that mice exposed to 2–20 kHz sound for 2 min at intensities of 100 dB SPL and greater showed behavioral signatures consistent with pain-related behavior.

10.1523/ENEURO.0064-26.2026.f8-1 Figure 8-1 Percentage change from Baseline for each measurement used to generate Facial Grimace and Body Position Scores for Sound- and No-Sound- exposed mice. **A**) Graphs of Facial Grimace (left) and Body Position (right) parameters for sound intervals of the interleaved sound exposure experiment (N = 17). Each dot is the average of one mouse for the indicated time-period after being transformed into a percentage compared to the baseline time-period. Sound time-periods have been reordered according to the increasing sound level. If a measurement resulted in a positive change from baseline (Ear Ratio, Ear Position, Rel. Nose Tip Position, Face Inclination), it was inverted so the percentage change from baseline associated with pain was a negative value. Box and whiskers have a line at the median and the box is the interquartile range with the whiskers extending to the maximum and minimum value of the data. Ear Ratio: repeated measures mixed effects model, p = 0.0014; post hoc comparisons using Tukey’s correction; baseline compared to 70 dB, **, p = 0.0063, baseline compared to 90 dB, *, p = 0.0149, baseline compared to 100 dB, *, p = 0.0217, baseline compared to 110 dB, *, p = 0.0104, baseline compared to 120 dB, **, p = 0.0036. Ear Position: repeated measures mixed effects model, p=<0.0001; baseline compared to 110 dB, ***, p = 0.0001, baseline compared to 120 dB, **, p = 0.001. Ear Tip Tilt: repeated measures mixed effects model, p = 0.0139. Eye Ratio: repeated measures mixed effects model, p = 0.0469. Rel. Nose Tip Position: repeated measures mixed effects model, p = 0.002; post hoc comparisons using Tukey’s correction; baseline compared to 90 dB, *, p = 0.0229, baseline compared to 100 dB, *, p = 0.0132, baseline compared to 110 dB, *, p = 0.0172. Mouth Position, Snout Position, Percent in Top, Vertical Line Crosses, and Face Inclination showed no significant differences between sound levels or from baseline. **B**) Graphs of Facial Grimace (left) and Body Position (right) parameters for no sound intervals of the interleaved sound exposure experiment (N = 17). Each dot is the average of one mouse for the indicated time-period after being transformed into a percentage compared to the baseline time-period. No sound time-periods have been reordered according to the preceding sound level. If a measurement resulted in a positive change from baseline (Ear Ratio, Ear Position, Rel. Nose Tip Position, Face Inclination), it was inverted so the percentage change from baseline associated with pain was a negative value. Box and whiskers have a line at the median and the box is the interquartile range with the whiskers extending to the maximum and minimum value of the data. Ear Ratio: repeated measures mixed effects model, p = 0.0072; Rel. Nose Tip Position: repeated measures mixed effects model, p = 0.0003, post hoc comparisons using Tukey’s correction; baseline compared to post 120 dB, **, p = 0.005. Ear Position, Ear Tip Tilt, Eye Ratio, Mouth Position, Snout position, Percent in Top, Vertical line Crosses, and Face Inclination showed no significant differences between no sound periods or from baseline. Download Figure 8-1, TIF file.

Composite scores for *No Sound* had no significant effects from the mixed model analyses for facial grimace or body position (Fig. 8*B*). It is noteworthy that during the period of no sound following 120 dB SPL, the Body Position Score surpassed the migraine pain-related behavior Level 2 threshold (Fig. 8*B*, dashed line). This change in behavior following 120 dB SPL is consistent with statistically significant changes observed in individual measurements described above (Fig. 7, Extended Data Fig. 8-1).

### The contribution of cochlear mechanotransduction to generating sound-evoked pain-related behavior

Pain caused by loud sounds could result from stress forces on auditory structures peripheral to the cochlea, such as sound pressure activating stretch receptors in the tympanic membrane or middle ear (Boedts, 2020). To test whether normal cochlear function is necessary for sound-evoked pain-related behavior, mice that lack mechanotransduction due to the loss the TMIE protein needed for proper function of the mechanotransduction channel in all cochlear hair cells (*Tmie*-knock-out mice, *Tmie^−/−^*) and littermate heterozygous or wild-type controls (*Tmie^CTL^*) were presented with the same interleaved sound protocol described above and tested for pain-related behavior (Gleason et al., 2009; Wu et al., 2017; Holt et al., 2021; Fettiplace et al., 2022). Importantly, both heterozygous and wild-type mice show normal cochlear mechanotransduction and thus can serve as valid controls (Bottom et al., 2024). These mice are maintained on a C57BL/6J background.

### Mice lacking TMIE largely showed no changes in individual behavioral measurements in response to sound exposure

**Figure 9.**
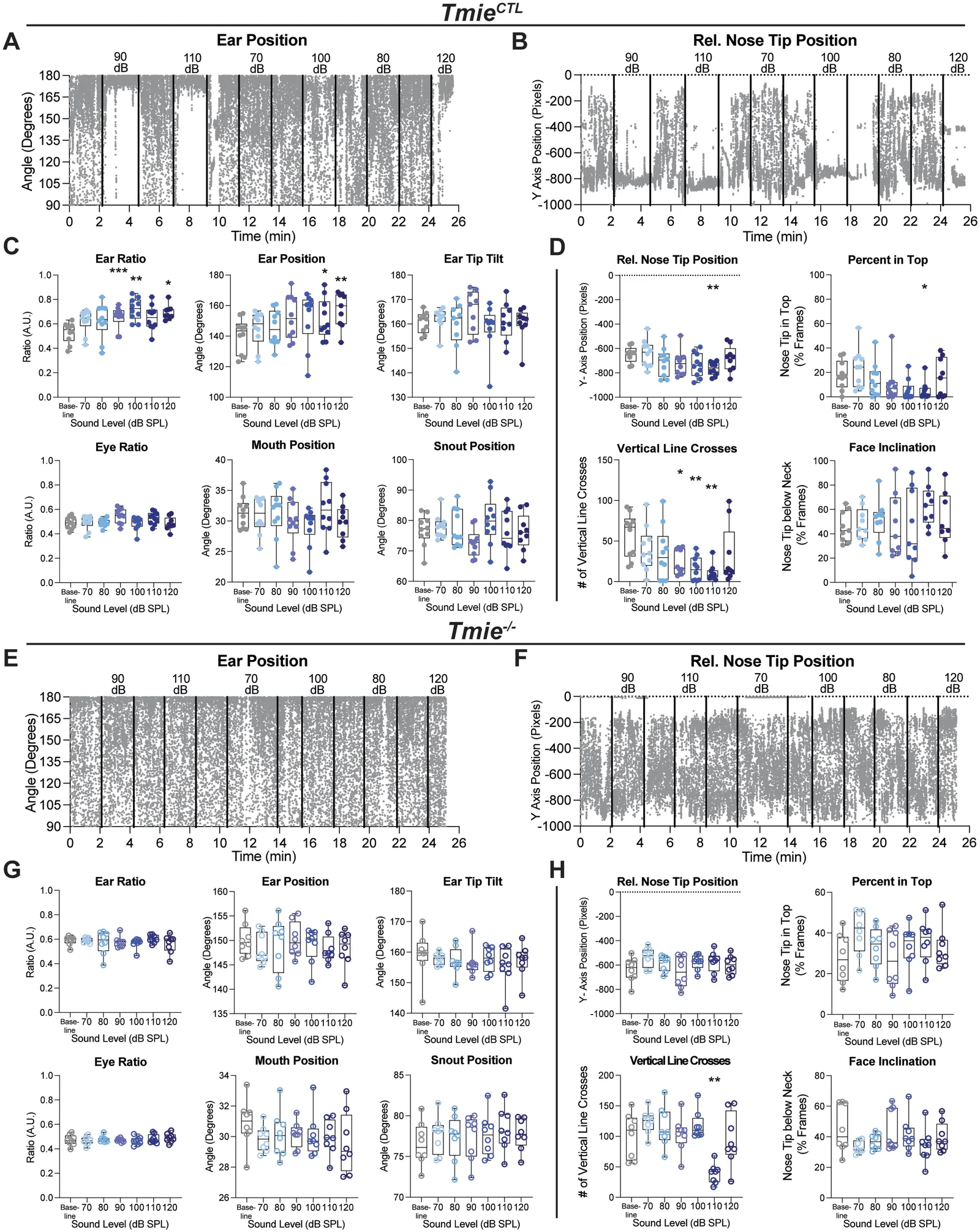
Mice lacking TMIE largely showed no changes in individual behavioral measurements in response to sound exposure. ***A***, Example of analyzed frames of Ear Position measurements for one *Tmie^CTL^* mouse for the entirety of one recording. The black vertical lines indicate when the sound levels were manually changed on or off. ***B***, Example of analyzed frames of Rel. Nose Tip Position measurements for one *Tmie^CTL^* mouse for the entirety of one recording. The black vertical lines indicate when the sound levels were manually changed on or off. ***C***, The following graphs show the averaged data for each facial parameter indicated. Each dot is the averaged data for the indicated 2 min time period for one *Tmie^CTL^* mouse (*N* = 10). The sound levels have been reordered from the lowest to the most intense sound level. Box and whiskers have a line at the median, and the box is the interquartile range with the whiskers extending to the maximum and minimum value of the data. Ear Ratio: repeated-measures mixed-effects model, *p* = 0.0009; post hoc comparisons using Tukey's correction; baseline compared with 90 dB; ***p = 0.0009; baseline compared with 100 dB; **p = 0.0049; baseline compared with 120 dB; *p = 0.0272. Ear Position, repeated-measures mixed-effects model, *p* = 0.0245; post hoc comparisons using Tukey's correction; baseline compared with 110 dB; *p = 0.027; baseline compared with 120 dB; **p = 0.0033. Eye Ratio, repeated-measures mixed-effects model, *p* = 0.0293. ***D***, The following graphs show the averaged data for each body position parameter indicated by the title. Each dot is the averaged data for the indicated 2 min time period for one *Tmie^CTL^* mouse (*N* = 10). The sound levels have been reordered from the lowest to the most intense sound level. Box and whiskers have a line at the median, and the box is the interquartile range with the whiskers extending to the maximum and minimum value of the data. Rel. Nose Tip Position, repeated-measures mixed-effects model, *p* = 0.014; post hoc comparisons using Tukey's correction; baseline compared with 110 dB; **p = 0.037. Percent in Top, repeated-measures mixed-effects model, *p* = 0.0124; post hoc comparisons using Tukey's correction, baseline compared with 110 dB; *p = 0.0224. Vertical Line Crosses, repeated-measures mixed-effects model, *p* = 0.0078; post hoc comparisons using Tukey's correction; baseline compared with 90 dB; *p = 0.0207; baseline compared with 100 dB; **p = 0.0026; baseline compared with 110 dB; **p = 0.0027. Face Inclination, repeated-measures mixed-effects model; ns, *p* = 0.1787; post hoc comparisons using Tukey's correction; baseline compared with 110 dB; *p = 0.0316. ***E***, Example of analyzed frames of Ear Position measurements for one *Tmie^−/−^* mouse for the entirety of one recording. The black vertical lines indicate when the sound levels were manually changed on or off. ***F***, Example of analyzed frames of Rel. Nose Tip Position measurements for one *Tmie^−/−^* mouse for the entirety of one recording. The black vertical lines indicate when the sound levels were manually changed on or off. ***G***, The following graphs show the averaged data for each facial parameter indicated. Each dot is the averaged data for the indicated 2 min time period for one *Tmie^−/−^* mouse (*N* = 8). The sound levels have been reordered from lowest to most intense sound level. Box and whiskers have a line at the median, and the box is the interquartile range with the whiskers extending to the maximum and minimum value of the data. ***H***, The following graphs show the averaged data for each body position parameter indicated by the title. Each dot is the averaged data for the indicated 2 min time period for one *Tmie^−/−^* mouse (*N* = 8). The sound levels have been reordered from the lowest to the most intense sound level. Box and whiskers have a line at the median, and the box is the interquartile range with the whiskers extending to the maximum and minimum value of the data. Rel. Nose Tip Position: repeated-measures mixed-effects model, *p* = 0.0401. Note this relationship shows an increase Rel. Nose Tip Position not associated with pain. Vertical Line Crosses: repeated-measures mixed-effects model, *p* = 0.0005; post hoc comparisons using Tukey's correction; baseline compared with 110 dB; **p = 0.0028. For extended data showing percentage change from baseline for each parameter, see Extended Data Figure 9-1.

Individual measurements of facial grimace and body position in *Tmie^CTL^* mice (Fig. 9) showed similar trends to those of the wild-type C57BL/6J mice described above. Example diary plots from an individual *Tmie^CTL^* mouse demonstrate changes in Ear Position and Relative Nose Tip Position during sound exposures (Fig. 9*A*,*B*). For data from all mice combined, for facial grimace measurements, Ear Ratio and Ear Position were significantly different from baseline at multiple sound levels (Fig. 9*C*). Specifically, significant increases were found in Ear Ratio (repeated-measures mixed-effects model, *p* = 0.0009; post hoc comparisons using Tukey's correction; 90 dB vs *Baseline* median difference = 0.127 AU; *p* < 0.001, 100 dB vs *Baseline* median difference = 0.173 AU; *p* < 0.01; and 120 dB vs *Baseline* median difference = 0.117 AU; *p* < 0.05) and in Ear Position (repeated-measures mixed-effects model, *p* = 0.0245; post hoc comparisons using Tukey's correction; 110 dB vs *Baseline* median difference = 3.1°; *p* < 0.05; and 120 dB vs *Baseline* median difference = 16.5°; *p* < 0.01). Eye Ratio showed a significant group effect but no post hoc tests were significantly different from baseline (repeated-measures mixed-effects model, *p* = 0.0293). For body position measurements, significant reductions were found in Relative Nose Tip Position, Percent in Top, and Vertical Line Crosses (Fig. 9*D*). Specifically, Relative Nose Tip Position (repeated-measures mixed-effects model, *p* = 0.014; post hoc comparisons using Tukey's correction; 110 dB vs *Baseline* median difference = −129 pixels; *p* < 0.01), Percent in Top (repeated-measures mixed-effects model, *p* = 0.0124; post hoc comparisons using Tukey's correction; 110 dB vs *Baseline* median difference = −15.1%; *p* < 0.05), and Vertical Line Crosses (repeated-measures mixed-effects model, *p* = 0.0078; post hoc comparisons using Tukey's correction; 90 dB vs *Baseline* median difference = −51 incidences; *p* < 0.05; 100 dB vs *Baseline* median difference = −53 incidences; *p* < 0.01; 110 dB vs *Baseline* median difference = −60 incidences; *p* < 0.01) all showed significant decreases at 110 dB SPL (Movie 4).

10.1523/ENEURO.0064-26.2026.f9-1 Figure 9-1 Percentage change from Baseline for each measurement used to generate Facial Grimace and Body Position Scores for *Tmie^CTL^* and *Tmie^-/-^* sound-exposed mice **A**) Graphs of Facial Grimace (left) and Body Position (right) parameters for sound intervals of *Tmie^CTL^* mice in the interleaved sound exposure experiment (N = 10). Each dot is the average of one mouse for the indicated time-period after being transformed into a percentage compared to the baseline time-period. Sound time-periods have been reordered according to increasing sound level. If a measurement resulted in a positive change from baseline (Ear Ratio, Ear Position, Rel. Nose Tip Position, Face Inclination), it was inverted so the percentage change from baseline associated with pain was a negative value. Box and whiskers have a line at the median and the box is the interquartile range with the whiskers extending to the maximum and minimum value of the data. Ear Ratio, *Tmie^CTL^*: repeated measures mixed effects model, p = 0.0025; post hoc comparisons using Tukey’s correction, baseline compared to 90 dB, **, p = 0.0017, baseline compared to 100 dB, **, p = 0.0092. Ear Position, *Tmie^CTL^*: repeated measures mixed effects model, p = 0.0349; post hoc comparisons using Tukey’s correction, baseline compared to 110 dB, *, p = 0.0237, baseline compared to 120 dB, **, p = 0.0035. Eye Ratio, *Tmie^CTL^*: repeated measures mixed effects model, p = 0.0321. Rel. Nose Tip Position, *Tmie^CTL^*: repeated measures mixed effects model, p = 0.0192; post hoc comparisons using Tukey’s correction, baseline compared to 110 dB, **, p = 0.0065. Percent in Top, *Tmie^CTL^*: repeated measures mixed effects model, p = 0.0064; post hoc comparisons using Tukey’s correction, baseline compared to 100 dB, **, p = 0.0035, baseline compared to 110 dB, ***, p = 0.0001. Vertical line crosses, *Tmie^CTL^*: repeated measures mixed effects model, p = 0.0062; post hoc comparisons using Tukey’s correction, baseline compared to 100 dB, ***, p = 0.0004, baseline compared to 110 dB, ****, p=<0.0001. Face Inclination **B**) Graphs of Facial Grimace (left) and Body Position (right) parameters for sound intervals of *Tmie^-/-^* mice in the interleaved sound exposure experiment (N = 8). Each dot is the average of one mouse for the indicated time-period after being transformed into a percentage compared to the baseline time-period. Sound time-periods have been reordered according to the increasing sound level. If a measurement resulted in a positive change from baseline (Ear Ratio, Ear Position, Rel. Nose Tip Position, Face Inclination), it was inverted so the percentage change from baseline associated with pain was a negative value. Box and whiskers have a line at the median and the box is the interquartile range with the whiskers extending to the maximum and minimum value of the data. Rel. Nose Tip Position *Tmie^-/-^*: repeated measures mixed effects model, p = 0.0293. Vertical Line Crosses, *Tmie^-/-^*: repeated measures mixed effects model, p = 0.0135; post hoc comparisons using Tukey’s correction, baseline compared to 110 dB, ****, p=<0.0001. Download Figure 9-1, TIF file.

**Movie 4.** *Tmie^CTL^* example video. Ten-second video clips from an original recording of the *Baseline* and 110 dB SPL time periods of a *Tmie^CTL^* mouse undergoing the interleaved sound protocol with sound included. The original clips are followed by the corresponding labeled video. The labels were generated by DLC, and any marker above 0.6 likelihood is shown for visualization. Only markers with a likelihood above 0.95 were used for analysis. [View online]

In contrast, in *Tmie^−/−^* mice, which lack functional cochlear mechanotransduction, most individual facial grimace measurements were not significantly different from baseline in any condition, except for one body position measurement that did show significant differences versus baseline (detailed below). Example diary plots of Ear Position and Relative Nose Tip Position from an individual mouse show no obvious behavioral changes during any sound exposure (Fig. 9*E*,*F*). For data from all mice combined, individual facial grimace measurements did not show any changes (Fig. 9*G*). However, of the four body position measurements, only Vertical Line Crosses were significantly reduced versus baseline at 110 dB SPL (Fig. 9*H*; repeated-measures mixed-effects model, *p* = 0.0005; post hoc comparisons using Tukey's correction; 110 dB vs *Baseline* mean difference = −68 incidences; *p* < 0.01). It is unclear what could cause the decreased turning observed here; however it is noteworthy that *Tmie*^−/−^ mice have altered vestibular function that causes repeated turning behavior, which caused a twofold increase in Vertical Line Crosses at baseline versus *Tmie^CTL^* mice (Fig. 9*D*,*H*). Relative Nose Tip Position also showed a significant group effect (repeated-measures mixed-effects model, *p* = 0.04), but the direction of this effect was toward increased position and thus was not suggestive of pain-related behavior.

### Cochlear mechanotransduction is required to generate sound-evoked pain-related behavior

**Figure 10.**
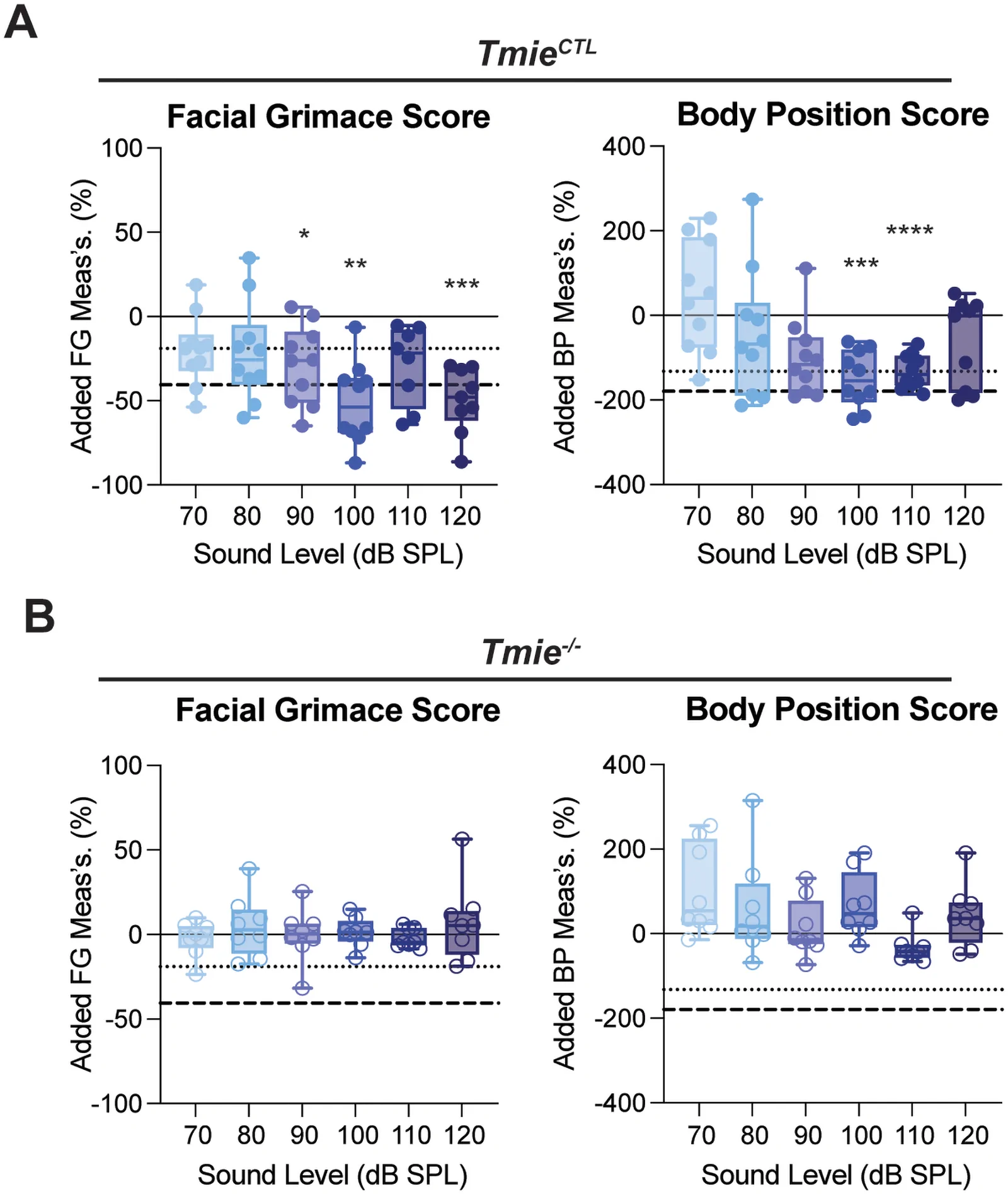
Cochlear mechanotransduction is required to generate sound-evoked pain-related behavior. Mice deficient in mechanotransduction do not show pain-associated behavioral changes when exposed to loud sounds like wild-type mice. ***A***, Facial Grimace (left) and Body Position (right) Scores for *Tmie^CTL^* mice exposed to sound (*N* = 10). Box and whiskers have a line at the median, and the box is the interquartile range with the whiskers extending to the maximum and minimum value of the data. Facial Grimace Score, *Tmie^CTL^*, repeated-measures mixed-effects model, *p* = 0.0004; post hoc comparisons using Tukey's correction, baseline compared with 90 dB; *p = 0.0417; baseline compared with 100 dB; **p = 0.0011; baseline compared with 120 dB; ***p = 0.0007. Body Position Score, *Tmie^CTL^*: repeated-measures mixed-effects model, *p* = 0.0031; post hoc comparisons using Tukey's correction; baseline compared with 100 dB; ***p = 0.0007; baseline compared with 110 dB; ****p = <0.0001. ***B***, Facial Grimace (left) and Body Position (right) Scores for *Tmie^−/−^* mice exposed to sound (*N* = 8). Facial Grimace Score, *Tmie^−/−^*, repeated-measures mixed-effects model; ns, *p* = 0.535; Body Position Score, *Tmie^−/−^*, repeated-measures mixed-effects model, *p* = 0.0296. For a mouse to be experiencing pain, the scores must be lower than −19.04 for Facial Grimace and −132.4 for Body Position (dotted line). For a mouse to experience Pain Level 2, the scores must be lower than −40.62 for Facial Grimace and −179.5 for Body Position (dashed line).

Individual measurements of both facial grimace and body position were converted into percentage change in baseline (Extended Data Fig. 9-1) and summed to generate composite scores (Fig. 10). Facial Grimace and Body Position Scores revealed sound-evoked pain-related behavior in *Tmie^CTL^* mice emerging at 100 dB SPL and greater sound intensities (Fig. 10*A*) replicating the findings from C57BL/6J detailed above. Specifically, Facial Grimace Score and Body Position Score both showed significant changes at 100 and 110 dB SPL consistent with normal hearing C57BL/6J mice presented above (Fig. 5; Facial Grimace Score, repeated-measures mixed-effects model, *p* = 0.0004; post hoc comparisons using Tukey's correction; 90 dB vs *Baseline* median difference = −26.0%; *p* < 0.05; 100 dB vs *Baseline* median difference = −53.7%; *p* < 0.01; 120 dB vs *Baseline* median difference = −47.9%; *p* < 0.001; Body Position Score, repeated-measures mixed-effects model, *p* = 0.0031; post hoc comparisons using Tukey's correction; 100 dB vs *Baseline* median difference = −154.8%; *p* < 0.001; 110 dB vs *Baseline* median difference = −140%; *p* < 0.0001).

In *Tmie^−/−^* mice, no significant differences were observed for Facial Grimace or Body Positions Scores at any sound intensity (Fig. 10*B*; Movie 5) suggesting that normal cochlear mechanotransduction is necessary for inducing sound-evoked pain-related behavior. These results suggest that sound pressure causing the activation of stretch receptors in peripheral auditory structures was not the source of sound-evoked pain-related behavior as measured and defined here (Rockley and Hawke, 1992; Rinaldi et al., 2013; Fournier et al., 2022). However, additional mechanisms that require normal cochlear function to generate subsequent pathophysiological responses cannot be ruled out, such as descending motor fibers innervating peripheral structures (such as cranial nerve V innervation of the tensor tympani), which could become hyperactive due to prior sound exposures and thereby could contribute to sound-evoked pain-related behavior (Westcott et al., 2013; Fournier et al., 2022; Sutton et al., 2025).

**Movie 5.** *Tmie^−/−^* example video. Ten-second video clips from an original recording of the *Baseline* and 110 dB SPL time periods of a *Tmie^−/−^* mouse undergoing the interleaved sound protocol with sound included. The original clips are followed by the corresponding labeled video. The labels were generated by DLC, and any marker above 0.6 likelihood is shown for visualization. Only markers with a likelihood above 0.95 were used for analysis. [View online]

In summary, this study demonstrates the feasibility of using a single camera angle to measure facial grimace and additional positional information from video recordings of mice to detect behavioral changes associated with persistent pain. Validation of this approach with a known painful stimulus (CGRP-induced migraine) and its application to an experimental condition (loud sound) allowed for the investigation of sound-evoked pain-related behavior in mice. Future studies could apply this method to other persistent pain conditions or to investigate further the sources and mechanisms responsible for pain-related behavior caused by sound exposure.

## Discussion

To gain a better understanding how sound-evoked pain-related behavior is generated and processed, it is crucial to implement methodology that allow for unbiased measuring of when sound becomes painful. Sound-evoked pain-related behavior was quantified using a deep neural network model trained to automatically place points on the face and body of mice that were video recorded while being presented with different intensities of sound. Using this method, changes in facial grimace (Langford et al., 2010; Tuttle et al., 2018; Chiang et al., 2022; Le Moëne and Larsson, 2023; McCoy et al., 2024) and body position (Filippidis et al., 2012; Kolstad et al., 2012; Vuralli et al., 2019; Weinreb et al., 2024; Ashiquzzaman et al., 2025) were analyzed to create an inventory capable of detecting pain-related behaviors in mice. CGRP injection to induce migraine in mice is a well-established persistent pain model (Rea et al., 2018) that was used to validate this method. CGRP-induced migraine caused detectable changes in behavior compared with baseline, supporting the approaches' applicability to study other forms of persistent pain. Sounds presented at intensities of 100 dB and above resulted in significant pain-related behavioral changes compared with baseline that appeared in both facial grimace and body position composite scores but were not present in mice lacking cochlear mechanotransduction. Taken together, these findings demonstrate that machine learning-based detection of pain-related behaviors during persistent painful states, such as CGRP-induced migraine and sound, can be reliably measured in mice.

### Machine learning-based automated detection of pain-related behaviors

The approach presented above used video recordings of freely moving mice during painful stimuli, namely, migraines and loud sound presentations, that were analyzed using a deep neural network model trained to automatically place points on the face and body of the mice. These points were used to calculate various individual parameters of facial grimace (Fig. 1) and body position (Fig. 3) that changed during painful stimuli (e.g., migraine) compared with baseline behavior and were plotted both as raw values (Figs. 2, 4) and as a normalized percentage change from baseline (Extended Data Fig. 5-1*A*).

The body position measurements in this method served two primary purposes: (1) to provide an additional metric of movement that complements the facial grimace score and (2) to demonstrate the possibilities of quantifying data from one to two *x*–*y* coordinate points using just one camera. By analyzing the *y*-axis of the nose tip, the average position could be calculated over a specified period (Relative Nose Tip Position). In this instance, when the animal moves up and down within the chamber, the average *y* value is higher compared with when the animal remains stationary in a hunched posture (Fig. 4). Additionally, by introducing horizontal or vertical boundaries within the video frames, it was possible to quantify the average amount of time the animal spent in a specific region of the frame (e.g., Percent in Top) or the frequency of crossing a defined boundary (e.g., Vertical Line Crosses). Furthermore, the relationship between the *y* values of two points could be analyzed to calculate the percentage of time this relationship was maintained (Face Inclination). These measurements provide examples of how pose estimation marker data can be utilized.

The percentage change from baseline for each facial grimace and body position parameter were separately summed to create two composite scores (Fig. 5) to detect changes in behavior associated with CGRP-induced migraine pain. Since injections of CGRP intraperitoneally in mice produce a migraine with a well-documented time course (Rea et al., 2018), where the peak of the pain occurs ∼30 min after injection, video recordings were performed from *t* = 20–40 min following injection. Strikingly, an empirical peak emerged from the analysis of these videos centered around the expected peak of 30 min, where different magnitudes of changes in pain-related behaviors were observed from *t* = 27–32 min compared with the remainder of the recording period before and after the peak. The median of each of these two periods, above referred to as “*Peak*” and “*Non-Peak*,” both differed significantly from baseline as well as from each other allowing for the delineation of two thresholds of migraine pain-related behavior (Levels 1, *Non-Peak*, and 2, *Peak*; Fig. 5*C*,*D*). The ability of the approach to delineate two pain-related behavioral levels provides an advancement for pain measurement.

### Sound presentation evokes pain-related behaviors at and above 100 dB SPL

Humans can experience pain when exposed to loud sounds at 120 dB and above (Yost and Killion, 1997). However, the sound exposure levels and protocols that might cause loudness intolerances in laboratory mice are not well known. With that in mind, suprathreshold levels of sound from 70 to 120 dB were used to probe whether mice would display behavioral changes associated with pain at any of these levels of sound using the now validated method of machine learning-based detection of pain-related behaviors. Sound was presented for 2-min-long periods and periods of silence (“*No Sound*”) occurred in between each sound presentation (Fig. 6). The purpose of the “*No Sound*” periods was to prevent an adaptation effect by continuous sound presentation and to serve as a control that behavioral changes restricted to sound presentation were reversible, which was true except for 120 dB SPL (Figs. 7, 8). Future iterations of the sound assay will include randomization of the sound levels up to 110 dB to rule out any effects of learning or adaptation on behavioral measures. However, the sound presentations in this study were novel to each mouse and only performed once. Both Facial Grimace and Body Position Scores were significantly changed from baseline starting at 100 dB SPL. It needs to be kept in mind that the determined “pain-related behavioral thresholds” are specific to the chosen experimental setting including the sound protocol and mouse model and have to be reexamined whenever used under different conditions. The strength of the approach does not lie in the absolute measured threshold levels but in the examination of a specific mouse model/condition, in comparison with the control conditions measured in the same settings.

### Probing auditory structures responsible for sound-evoked pain-related behavior using *Tmie*^−/−^ mice

It remains unclear which peripheral or central auditory structures are necessary for sound-evoked pain. Multiple theories exist for the anatomical sources of sound-evoked pain (Radziwon and Salvi, 2020; Salvi et al., 2022; Smith et al., 2025), most of which posits that the process is initiated by pathological changes in the function of peripheral auditory structures that ultimately alter the function of central auditory processing, resulting in enhanced efferent activation of the tensor tympani muscle (Mukerji et al., 2010; Boedts, 2020; Sutton et al., 2025), hyperactivity of type II auditory nerve fibers which may act as auditory nociceptors (Flores et al., 2015; Liu et al., 2015; Nowak et al., 2021), and/or hypoactivation of the auditory cortex and concurrent increased activity in the thalamus (Song et al., 2014; Zhou et al., 2022; Clayton et al., 2024). However, it is conceivable that sound-evoked pain, especially when caused by loud sounds, may not rely on cochlear mechanotransduction in the inner ear but rather could be caused by the activation of stretch receptors in the middle ear or on the tympanic membrane that could be triggered at high pressure levels to induce pain due to sound (Nagai and Tono, 1989; Rockley and Hawke, 1992; Rinaldi et al., 2013; Fournier et al., 2022).

*Tmie*^−/−^ mice (Gleason et al., 2009; Wu et al., 2017; Holt et al., 2021; Fettiplace et al., 2022; Bottom et al., 2024) exhibit normal development of outer and middle ear but have profound inner ear sensory dysfunction and were therefore here used to investigate the contributions of peripheral auditory structures to sound-evoked pain as measured using the method described above. *Tmie*^−/−^ mice showed no significant behavioral changes at any sound exposure level that reached the pain-related behavioral threshold for either the Facial Grimace or Body Position Scores (Figs. 9, 10). The littermate-control mice (*Tmie^CTL^*) exhibited similar behavioral changes associated with sound presentation as the wild-type C57BL/6J mice previously shown, indicating pain-related perception during loud sound exposure. Thus, cochlear function in *Tmie^CTL^* was necessary for a normally hearing animal to display sound-evoked pain-related behavior. It is still possible that in animals with disordered hearing or changes to the middle ear (such as otitis media), the middle ear could play a role in auditory pain perception. As in tinnitus and paralleled in phantom limb syndrome (Limakatso et al., 2020), central processing of auditory pain also likely plays a role in auditory pain disorders, such as pain hyperacusis (McGill et al., 2022); however, sound detection by the peripheral auditory system appears to be necessary for the generation of auditory pain-related behavior in response to loud sounds in a normally hearing mouse.

### Implications for animal noise exposure protocols

Loud sound exposures are frequently used to study noise-induced hearing loss (NIHL) in animals. Specifically, loud sound protocols are used to induce temporary or permanent hearing damage to cochlear function as tested by ABR thresholds and morphological integrity of the cochlea (hair cell loss; synaptopathy; Fernandez et al., 2020; Qian et al., 2024). Such protocols often use 2 h of 110 dB SPL (or greater) sound, exceeding the levels causing pain behavior as reported here (Nowak et al., 2021; Wood et al., 2021). It is noteworthy that similar noise exposures have been shown to cause variable degrees of cochlear damage across institutions and mouse strains (Schrode et al., 2022), suggesting that pain-related behavioral responses may also differ under these conditions. While intense noise levels are necessary to address specific research questions related to NIHL, the findings presented here highlight the possibility of additional behavioral effects accompanying such exposures.

### Expanding existing methods for detecting auditory-evoked behavior

Several methods exist to measure sound-evoked behavior in rodents. These methods fall into two categories: (1) measurements of reflexive responses to sound and (2) measurements of aversion to sound. Acoustic startle responses and subsecond facial responses model loudness perception (Manohar et al., 2023; Clayton et al., 2024), while conditioned aversion assays measure the preference of a mouse for a well-lit chamber over a dark chamber with noise going against the animal's instinct (Manohar et al., 2017, 2023). Thus, loudness hyperacusis can be modeled and quantified in animals. The results of reaction time and conditioned aversion assays have been interpreted as aversion to sound, but the conclusion that this is due to pain is unclear. Therefore, the subset of hyperacusis known as pain hyperacusis currently has no specific animal model. A tool to measure behavioral changes associated with pain-related behavior caused by sound, as presented here, is needed to test whether mice can effectively model the subclasses of pain hyperacusis observed in patients.

### Future applications and modifications of detecting pain-related behavior with composite Facial Grimace and Body Position Scores

The composite Facial Grimace and Body Position Scores presented here provide a valuable initial framework for quantifying pain-related behavioral changes using an unweighted summation of metrics. Notably, certain parameters, such as Ear Ratio and Ear Tip Tilt, exhibited minimal changes in magnitude but significant reductions in variation during painful stimuli, highlighting their potential relevance. Future studies should evaluate the relative contributions of individual parameters in specific pain contexts, allowing for refinement through inclusion, exclusion, or weighting of metrics to optimize the scoring system. It is important to note that the scoring method is designed to provide an inventory that is designed to accommodate further expansion, including new measurements and behavioral categories, such as complex behaviors (e.g., grooming, rearing, exploring, and respiratory rate), which are associated with persistent painful states (National Research Council (US) Committee on Recognition and Alleviation of Pain in Laboratory Animals, 2010; Turner et al., 2019). Changes in complex behaviors were qualitatively observed in recordings of CGRP-induced migraine and sound exposure but not quantified here. Incorporating these behaviors into a potential third score may enhance the sensitivity and robustness of the method while keeping the simplicity of one camera angle for ease of implementation. By refining the inventory and selecting the most indicative metrics, this approach has the potential to generate a graded pain-related behavioral scale, as demonstrated here by its ability to distinguish between two levels of migraine pain-related behavior in this initial application.

## Synthesis

Reviewing Editor: David Schoppik, New York University Grossman School of Medicine Decisions are customarily a result of the Reviewing Editor and the peer reviewers coming together and discussing their recommendations until a consensus is reached. When revisions are invited, a fact-based synthesis statement explaining their decision and outlining what is needed to prepare a revision will be listed below. The following reviewer(s) agreed to reveal their identity: Bo Duan.

1. A major concern is the apparently small magnitude of changes in many parameters, despite statistical significance. This raises questions about biological relevance and whether the method detects robust pain signals or subtle, potentially non-specific variations. eNeuro recommends estimation statistics (see Instructions for Authors, which points to https://www.estimationstats.com/#/ as a starting point). We feel strongly that the manuscript should report effect sizes and/or confidence intervals so that it is possible for the readers to evaluate whether the differences are likely to reflect low signal-to-noise ratio, possibly due to variability in mouse positioning (e.g., profile view dependency, with gaps in diary plots from non-profile frames). Finally, there is an inconsistency in reporting medians versus means that should be fixed in the revision.

Specifically, the following points were noted:

-Facial Grimace Parameters: In the CGRP model, Eye Ratio and Mouth Position show consistent significance, with small absolute changes (e.g., Eye Ratio: -0.078 AU from baseline, representing ~10-15% relative change; Mouth Position: -2.98{degree sign} to -3.49{degree sign}). E

-Ear-related metrics (Ratio, Position, Tip Tilt) and Snout Position often lack significance or show minimal shifts (e.g., Ear Position medians differ by <5{degree sign} across conditions).

-In sound exposures, ear metrics drive changes (e.g., Ear Ratio: +0.036-0.093 AU at higher dB), but these are tiny in pixel/angle terms (e.g., 8-10{degree sign} for Ear Position at 110-120 dB).

-Composite Facial Grimace Scores amplify this (-20-40% from baseline), but reliance on summation masks individual parameter weakness-many contribute near-zero or inconsistent effects.

- Body Position Parameters: Similar issues.

2. Addition of scent holes on one side to encourage mice to remain mostly in profile was a nice idea. We recommend adding quantitative data showing that this approach does indeed keep animals in profile view for most of each recording.

3. How do the measured ratios and angles change as a function of mouse position (facing directly towards camera, full profile, and different suboptimal profile views)? The manuscript mentions that there are gaps in data when animals do not face the camera, which is understandable. The question, however, is the extent to which these measures and angles are invariant when animals are in a suboptimal viewing angle.

4. The core claim -- that observed behaviors reflect "pain" rather than non-painful auditory responses -- is not convincingly supported. Facial and body changes (e.g., ear flattening/rotation, reduced movement, hunching) overlap with startle reflexes, freezing, or orienting/attending to novel sounds, especially in an enclosed chamber with sudden onsets. The protocol (2-min broadband noise at 2-20 kHz) likely elicits these confounds, and the method averages over epochs without parsing transient vs. sustained responses. One way to further support that these noise evoked responses are due to pain would be to administer an analgesic, and show that responses are reversed. Key controls should be used, such as analgesic alone, as some drugs (like opioids) cause depressed behaviors at higher doses.

5. The manuscript defines pain thresholds based on CGRP-induced migraine. Then the authors used these thresholds to classify sound-evoked behaviors as pain. CGRP-induced migraine represents an internal, persistent pain state, while sound exposure is an external stimulus. We caution that these modalities may therefore not be directly comparable on a single behavioral scale.

6. The manuscript did not provide the details of the DeepLabCut-based analysis. We would like to see details about the tracking accuracy and error rates, and a discussion of the effects of the small training dataset (816 frames).

4. The sound stimuli are presented in a fixed order. This design introduces potential order effects, including sensitization or cochlear damage, which may influence the interpretation of subsequent responses. We would like to see this limitation discussed, or experiments repeated with random order.

Minor:

1. The manuscript did not report whether sex differences were examined, although both male and female mice were used.
